# Genome-wide distribution of 5-hydroxymethyluracil and chromatin accessibility in the *Breviolum minutum* genome

**DOI:** 10.1186/s13059-024-03261-3

**Published:** 2024-05-06

**Authors:** Georgi K. Marinov, Xinyi Chen, Matthew P. Swaffer, Tingting Xiang, Arthur R. Grossman, William J. Greenleaf

**Affiliations:** 1 Department of Genetics, Stanford University, Stanford, CA 94305 USA; 2 Department of Bioengineering, Stanford University, Stanford, CA 94305 USA; 3 Department of Biology, Stanford University, Stanford, CA 94305 USA; 4 Department of Plant Biology, Carnegie Institution for Science, Stanford, CA 94305 USA; 5 Center for Personal Dynamic Regulomes, Stanford University, Stanford, CA 94305 USA; 6 Department of Applied Physics, Stanford University, Stanford, CA 94305 USA; 7 Chan Zuckerberg Biohub, San Francisco, CA USA

## Abstract

**Background:**

In dinoflagellates, a unique and extremely divergent genomic and nuclear organization has evolved. The highly unusual features of dinoflagellate nuclei and genomes include permanently condensed liquid crystalline chromosomes, primarily packaged by proteins other than histones, genes organized in very long unidirectional gene arrays, a general absence of transcriptional regulation, high abundance of the otherwise very rare DNA modification 5-hydroxymethyluracil (5-hmU), and many others. While most of these fascinating properties are originally identified in the 1970s and 1980s, they have not yet been investigated using modern genomic tools.

**Results:**

In this work, we address some of the outstanding questions regarding dinoflagellate genome organization by mapping the genome-wide distribution of 5-hmU (using both immunoprecipitation-based and basepair-resolution chemical mapping approaches) and of chromatin accessibility in the genome of the Symbiodiniaceae dinoflagellate *Breviolum minutum*. We find that the 5-hmU modification is preferentially enriched over certain classes of repetitive elements, often coincides with the boundaries between gene arrays, and is generally correlated with decreased chromatin accessibility, the latter otherwise being largely uniform along the genome. We discuss the potential roles of 5-hmU in the functional organization of dinoflagellate genomes and its relationship to the transcriptional landscape of gene arrays.

**Conclusions:**

Our results provide the first window into the 5-hmU and chromatin accessibility landscapes in dinoflagellates.

**Supplementary Information:**

The online version contains supplementary material available at 10.1186/s13059-024-03261-3.

## Background

Dinoflagellates are perhaps the most remarkable lineage within the spectrum of known eukaryote diversity, with numerous extreme deviations from the genomic and cellular organization of other eukaryotes, especially regarding their highly unusual nuclei [1–6]. They are also a very diverse, successful, and ecologically important, primarily unicellular group that includes numerous photosynthetic lineages, free-living heterotrophs, and even parasites, playing a major ecological role in marine ecosystems. The best-known such example is the endosymbiotic association of Symbiodiniaceae dinoflagellates [7] with reef-building corals. The photosynthetic capability of the dinoflagellate symbionts provides the metabolic foundation for the highly biologically diverse reef ecosystems [8], and the expulsion of these symbionts from their host cells upon heat stress causes coral “bleaching” and the eventual death of coral reefs [9], an increasingly acute problem in the modern world due to the effects of global climate change [10].

The list of unorthodox features of dinoflagellate nuclei is long [1, 3, 4, 11–14]. Dinoflagellate chromosomes exist in a permanently condensed liquid crystalline state throughout most of the cell cycle and are characterized by an unusually low protein-to-DNA ratio (1:10, compared to 1:1 in other eukaryotes [2, 15]). This condensed, protein-poor structure is caused by the loss of nucleosomal histones as the main packaging component of chromatin. This role has instead been taken over by a distinct set of proteins — small dinoflagellate-specific virus-derived nucleoproteins (DVNP) and histone-like proteins (HLPs) [16–24] — that appear to have been acquired through horizontal gene transfer from viruses and bacteria, respectively [25, 26]. Such chromatin composition is an extreme departure from the norm for a eukaryote, as nucleosomal chromatin is otherwise universal [27]. Dinoflagellates have not lost histones — in fact, multiple and highly diverse histone genes are retained in all dinoflagellates for which genomic data is available [28] — but these histone proteins are extremely divergent from those of other eukaryotes and it is not clear what role these proteins might play in dinoflagellate nuclei. It is also an open question whether DVNPs and/or HLPs might provide similar levels of physical protection of DNA to nucleosomes. Past studies have suggested that DVNPs bind to DNA with similar affinity to histones [25] but HLPs, although they can compact DNA in a concentration-dependent manner, have weaker affinity than histones [29–31]. However, whether and how these proteins confer distinct chromatin states through their association with DNA is not known.

Genome organization in dinoflagellates also represents a highly derived state, as their genes are organized into long unidirectional gene arrays [32–35], presumably transcribed as a single unit, and mature mRNAs generated through the addition of a spliced leader (SL) sequence [32, 36, 37] (*trans*-splicing). Transcriptional regulation is thought to be largely absent, with all genes transcribed at all times. The primary mode of gene regulation is presumed to be at the level of translation and/or RNA stability.

Dinoflagellates also contain the otherwise highly unusual for eukaryotes 5-hmU modification (present in abundance and described originally in some phages [38]), which was first discovered in the 1970s [39–41]. Unexpectedly large fractions of thymines (T) in the genome of various species were reported to be replaced by 5-hmU — 12% in *Exuviaella cassubica* [41] (synonym for *Prorocentrum cassubica*), 12% in *Symbiodinium microadriaticum* [41], 37–38% in *Crypthecodinium cohnii* [41, 42], 62% in *Amphidinium carterae* [41], 62.8% in *Prorocentrum micans* [43], and 68% in *Peridinium triquetrum* [41]. What functions 5-hmU might have is not known, but it has been suggested that it enhances the flexibility and hydrophilicity of double-stranded DNA [44], especially in some sequence contexts [45–47].

To this day very little is known about the inner workings of these remarkable organisms, and how the eukaryote nucleus has been transformed and adapted in such a dramatic way remains a major riddle. Recently, we and others [48, 49] began to unravel some of these mysteries by applying three-dimensional genome conformation mapping using Hi-C [50] to the members of Symbiodiniaceae *Breviolum minutum* and *Symbiodinium microadriaticum*, showing that the genome is folded into distinct topologically associating domains coinciding with pairs of divergent gene arrays and separated by the points where convergent gene arrays meet (termed “dinoTADs”, numbering ∼583 in *B. minutum*). These domains appear to be the product of strong transcription-induced supercoiling in a context of extremely long transcriptional units and the absence of histones.

Earlier, de Mendoza et al. [51] mapped the distribution of the frequently found in eukaryotes 5-methylcytosine (5mC) modification in two members of the Symbiodiniaceae — *Fugacium kawagutii* and *Breviolum (Symbiodinium) minutum*. An unusual compared to other eukaryotes pattern of uniform hypermethylation throughout the genome was observed.

In this work, we aim to answer two other still open questions — what the potential roles and genomic distribution of the 5-hmU modification in dinoflagellate genomes are, and what what the nature of the genome-wide chromatin accessibility landscape is. Whether dinoflagellate genomes are uniformly accessible given the lack of histones, whether distinct open chromatin regions exist as in conventional eukaryotes, and whether perhaps the inverse phenomenon is observed — localized areas of decreased accessibility — and if and how 5-hmU might relate to these properties, is an open question.

**Fig. 1:**
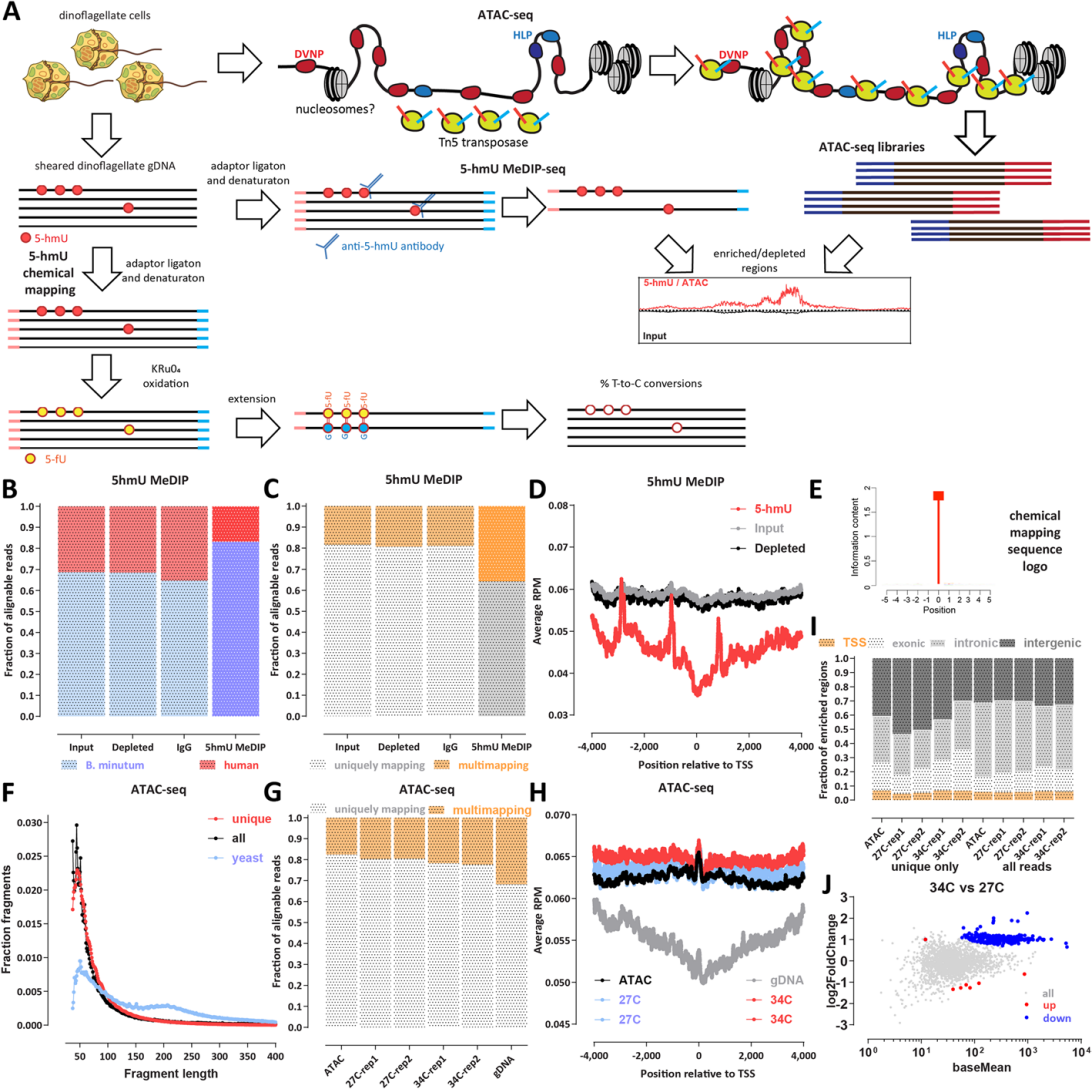
Mapping the 5-hmU and chromatin accessibility landscape in *B. minutum*. **A** Experimental outline. ATAC-seq maps accessible regions in the genome by taking advantage of the preference of the Tn5 transposase for inserting into physically accessible DNA. We mapped 5-hmU using two orthogonal approaches — MeDIP-seq, using an *α*5-hmU antibody, and chemical conversion, using oxidation of 5-hmU to 5-fU, eventually resulting in T-to-C base conversion in final libraries. **B** Proportions of human and *B. minutum* gDNA in 5-hmU Methylated DNA immunoprecipitation sequencing (MeDIP-seq) and control libraries. A mixture of human and dinoflagellate gDNA was used as input to MeDIP-seq experiments, and the fraction of reads that map to each genome is shown. The 5-hmU MeDIP-seq library is enriched for dinoflagellate reads confirming the specificity of 5-hmU pull down. **C** Proportion of multimapping reads in 5-hmU MeDIP-seq and control libraries. The 5-hmU MeDIP-seq library exhibits a higher fraction of multimapping reads, suggesting that 5-hmU is enriched over repetitive elements. **D** Metaprofiles of 5-hmU and control libraries signal over *B. minutum* transcription start sites/gene starts. **E** Basepair-resolution chemical mapping of 5-hmU does not reveal a sequence motif associated with the modification in *B. minutum*. Shown is the consensus sequence logo over modified T positions in the *B. minutum* genome as defined from out chemical mapping datasets. **F** A nucleosomal signature is not visible in the fragment length distribution of *B. minutum* ATAC-seq datasets. Shown are uniquely mapping reads alone as well as all reads that can be mapped. Also shown for comparison is the fragment length distribution for a representative yeast (*S. cerevisiae*) dataset. **G** Proportion of multimapping reads in *B. minutum* ATAC-seq datasets as well as a control genomic DNA (gDNA) library. Repetitive elements are depleted in ATAC-seq datasets. **H** Metaprofiles of ATAC-seq signal over *B. minutum* transcription start sites/gene starts as well as the gDNA control show no preferential accessibility over these regions. **I** Distribution of ATAC-seq regions of enrichment relative to annotated genomic features. **J** Differential accessibility analysis for the 27 °C and 34 °C conditions reveals little large-scale change in the global accessibility landscape

To answer these questions, we mapped chromatin accessibility and distribution of 5-hmU in the genome of *B. minutum* (Fig. 1A). We find that 5-hmU is enriched over certain repetitive element classes and often around the boundaries between gene arrays. In contrast, chromatin accessibility is anti-correlated with elevated 5-hmU levels; this inverse relationship is specifically strong around gene array/dinoTAD boundaries, pointing to potential localization of histones (or other proteins that protect DNA) to regions enriched for 5-hmU (and thus conferring them greater protection from transposase insertion). We do not detect increased accessibility associated with transcription start sites (TSSs), and generally, we do not observe strongly localized DNA accessibility peaks in the genome comparable to those in metazoans. These results provide a foundation for the future detailed understanding of the organization of transcription in dinoflagellates and its interplay with DNA modifications.

## Results

### Mapping 5-hmU and chromatin accessibility in *B. minutum*

In order to map the distribution of 5-hmU in the *B. minutum* genome, we first adapted the MeDIP (Methylated DNA ImmunoPrecipitation) protocol [52] for mapping DNA methylation using high-throughput sequencing (MeDIP-seq [53]; Fig. 1A). In MeDIP-seq, DNA is first sheared down to fragments of length 200–400 bp, then sequencing adaptors are ligated, followed by denaturation. Single-stranded DNA (ssDNA) is then subjected to pull down with an antibody against the targeted DNA modification, and the enriched DNA is PCR-amplified and sequenced. This provides a readout of general localized enrichment of the DNA modification in question along the genome, but without providing basepair-resolution of modification levels.

We used an antibody specific to 5-hmU (see the “ Methods” section) and a spike-in control to confirm the specific enrichment of 5hydoxymethyluracil. As mammalian genomes do not contain appreciable amounts of 5-hmU, we used a mixture of human and *B. minutum* genomic DNA (gDNA) as input to the MeDIP procedure, and we also sequenced three different controls — input DNA, “depleted” DNA (the supernatant remaining after the immunoprecipitation step), and an IgG control (using only beads with no primary antibody). We observed that the fraction of human reads decreased ∼2 × after 5-hmU MeDIP relative to controls (Fig. 1B), confirming the specific enrichment of dinoflagellate DNA. We also made an interesting observation — 5-hmU MeDIP is also ∼2 × enriched for multimapping reads compared to the controls (Fig. 1C). This suggests that 5-hmU is preferentially associated with repetitive and transposable elements, because multimapping reads (i.e., reads that map equally well to multiple locations in the genome) are usually primarily derived from such regions.

We did not observe enrichment or 5-hmU around the starting positions of genes (Fig. 1D) — in fact, we observe a slight depletion ± 1-kb around gene starts (note that the three spikes observed in the plot are an artefactual result due to the presence of collapsed repeats in the current *B. minutum* assembly; see further discussion on this topic below).

**Fig. 2:**
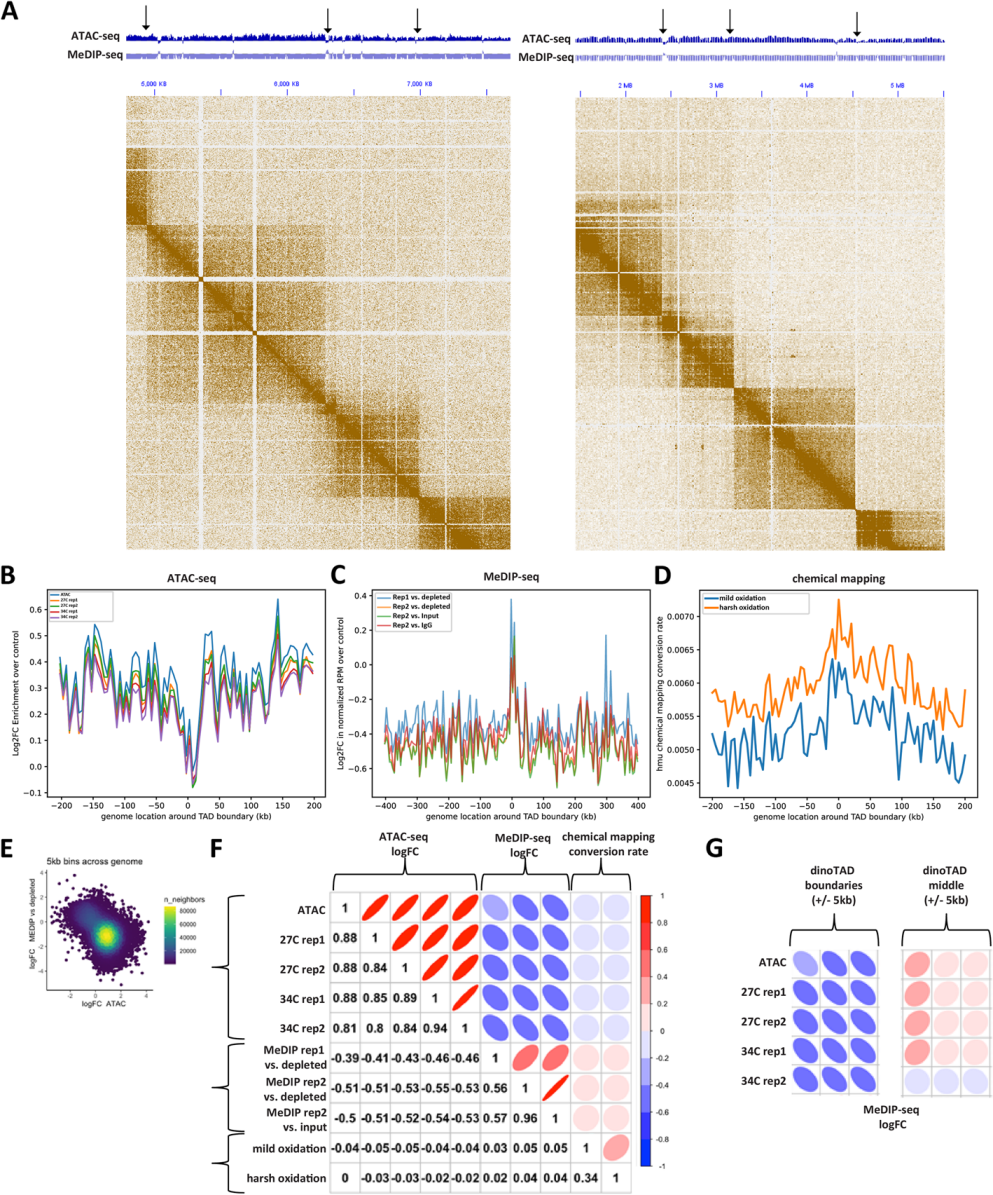
Inverse correlation between 5-hmU and chromatin accessibility and association with dinoTADs boundaries in the *B. minutum* genome. **A**, **B** Representative snapshots of the distribution of 5-hmU enrichment and decreased chromatin accessibility relative to dinoTAD boundaries. **C** Depletion of ATAC-seq signal around dinoTAD boundaries. **D** Enrichment of MeDIP-seq signal around dinoTAD boundaries. **E** Increased 5-hmU chemical mapping conversion rate around dinoTAD boundaries. **F**, **G** ATAC-seq and MeDIP-seq are generally anti-correlated (calculated for 5-kbp bins over the whole genome). **H** ATAC-seq and MeDIP-seq are specifically strongly anti-correlated around dinoTAD boundaries

We also deployed an orthogonal method for mapping 5-hmU at base-pair resolution using chemical conversion of 5hmU into cytosine C using a previously developed in the context of kinetoplastids protocol [54] (Fig. 1A; see the “ Methods” section for further details). The protocol involves the chemical oxidation of 5-hmU to 5-fU (5-formyluracil), which is carried out using treatment with KRuO_4_. Subsequently, ionized 5-fU can basepair with G instead of A, causing T-to-C conversion in final sequencing libraries. We carried out experiments using both “mild” and “harsh” oxidation conditions, with the latter exhibiting higher conversion rates as expected (Fig. 2D).

The archetypal DNA modification in eukaryotes is 5mC, which, especially in mammals and other traditional model systems, is found specifically in a CpG context. We asked whether there might be analogous sequence preferences for the deposition of 5-hmU in dinoflagellates. However, in contrast to 5mC in mammals, we do not find any sequence preference for T bases modified into 5-hmU in *B. minutum* (Fig. 1E). We note that early studies from the 1980s reported that 5-hmU preferentially replaces thymines in TA and TC sequence contexts [42], and we do not recover a strong such preference in our datasets; it is possible that such preferences indeed exist in *Crypthecodinium cohnii*, which was assayed by those studies, but not in Symbiodiniaceae, or that the discrepancy is due to methodological differences. It should also be noted that the current chemical mapping protocol we employed does not provide for a 100% conversion rate of 5-hmU modified bases; this means that we are able to evaluate relative basepair-resolution modification levels, but it is currently not possible to estimate the absolute levels of 5-hmU in the *B. minutum* genome and for any local region in it based on chemical mapping data alone.

To map the *B. minutum* chromatin accessibility landscape, we utilized ATAC-seq [55] (Assay of Transposase-Accessible Chromatin using sequencing), specifically in its omniATAC [56] modification (see Methods). The conceptual basis for ATACseq is the strong preference of the Tn5 transposase for inserting into physically accessible DNA. Open chromatin regions can thus be preferentially tagged with sequencing adapters in situ, followed by DNA isolation, PCR amplification, and sequencing. We note that in the context of dinoflagellates it is quite possible that the problem is the inverse to what it is in conventional eukaryotes — if the whole genome, or most of it, is devoid of nucleosomes, and DVNPs and HLPs do not provide similar levels of protection, Tn5 is expected to insert largely uniformly in it (Fig. 1A). We might instead be looking for regions of inaccessibility, i.e., decreased ATAC-seq signal, rather than preferentially accessible, enriched in ATAC-seq libraries regions, with these regions likely corresponding to the putative locations of nucleosomal association with DNA.

We generated a very deeply sequenced (∼130 million mapped reads) library from actively growing cells referred to as “ATAC” in subsequent figures) as well as two replicates each for cells grown at the usual temperature of 27 °C and heat-stressed cells that were incubated at 34 °C (“27C” and “34C,” respectively).

In eukaryotes with nucleosomal chromatin, ATAC-seq libraries sequenced in a paired-end format display a characteristic nucleosomal signature in their fragment length distribution, with a subnucleosomal peak at ≤ ∼120 bp, a prominent mononucleosomal peak, and a weaker dinucleosomal peak (an example for yeast is shown in (Fig. 1F)). In marked contrast, *B. minutum* ATAC-seq only displays a peak at short fragment lengths (∼60 bp), with no nucleosomal peaks (Fig. 1F). Thus, we conclude that wherever they are found in the genome, nucleosomes apparently are of too low abundance to substantially affect the overall fragment length distribution, while DVNPs and HLPs do not form structures consisting of multiple closely positioned proteins that strongly protect against transposition. We also observe a modest depletion of multimapping reads in ATAC-seq libraries relative to a matched naked gDNA control (Fig. 1G), i.e., the opposite trend of that observed for MeDIP-seq. ATAC-seq signal is also not enriched around gene start positions (Fig. 1H). This suggests that chromatin accessibility is reduced over repetitive elements, while there is no specific open chromatin structure similar to that of eukaryotes around promoter regions (that is, chromatin that is open relative to adjacent nucleosomal DNA).

Genome browser inspection of ATAC-seq and gDNA controls (Additional file 1: Supplementary Figs. 1 and 2) revealed that the available *B. minutum* assemblies (both the original and the Hi-C-scaffolded ones) include multiple collapsed repeats, i.e., regions that exist in multiple copies in the actual genome but are only present in the assembly as a single copy (or as many fewer copies than their actual abundance in the genome). This complicates the interpretation of sequencing datasets as these regions appear as artificial “peaks” if analysis is not carried out against a proper control. Therefore, we performed all subsequent analyses as a comparison against matched input or negative gDNA controls. The regions of enrichment over gDNA that we identified did not show a concentration around gene starts/TSSs (Fig. 1I)., and they show overall lower enrichment over background/controls than ATAC-seq peaks in human datasets [57] (Additional file 1: Supplementary Fig. 3), i.e., we do not really observe strongly localized chromatin accessibility as in other eukaryote genomes. Comparing the heat stressed (34 °C) and normal temperature (27 °C) conditions did not reveal large-scale changes in the chromatin accessibility landscape (Fig. 1J).

### Heterologous expression of DVNPs has a modest effect on chromatin organization in the yeast *S. cerevisiae*

Previous studies had examined the effect of DVNPs on chromatin structure by expressing a DVNP (*Hematodinium* sp. DVNP.5) in the yeast *Saccharomyces cerevisiae* [58]. The resulting changes in the chromatin landscape (measured using MNase-seq) were reported to reveal nucleosome disruption, while overall the expression of the DVNPs had a negative effect on cell growth, likely because it impaired transcription. We sought to replicate and expand on these results by expressing several DVNPs in *S. cerevisiae* and carrying out ATAC-seq as well as single-molecule footprinting (SMF [59, 60]). SMF uses a GpC methyltransferase (MTase), or a combination of a GpC MTase and a CpG MTase, in species where there is no endogenous 5mC methylation (which is the case in yeast) to enzymatically label accessible DNA (physically protected DNA is refractory to methylation). DNA is subsequently sheared and subjected to base conversion, allowing methylated bases to be read out in each individual molecule. This provides information about the absolute levels of accessibility/protection along the genome (which are measured as the fraction of reads that are methylated over each position, or, more commonly, the inverse of it), and we aimed to evaluate these properties in the context of DVNP occupancy).

We heterologously expressed (see the “ Methods” section) three different DVNPs in yeast — the previously assayed *Hematodinium* sp. DVNP.5 as well as *Hematodinium* sp. DVNP.12 and *B. minutum* DVNP symbB.v1.2.006931. We carried out ATAC-seq and SMF using an internal control in all experiments — *Candida glabrata* cells, which we used to account for experimental variation, as previously described [61] (because the efficiency of transposition or methylation can vary between reactions, if the goal is to compare global between different conditions, it is helpful to have a spike-in control whose properties are identical across all samples, and which can be thus used for normalization between them).

ATAC-seq did not reveal dramatic changes in the accessibility landscape upon DVNP expression (Additional file 1: Supplementary Figs. 5 and 4) except perhaps for a slight decrease in the height of some peaks. On the other hand, SMF data showed a decrease in accessibility around TSSs and reduced strength of nucleosome positioning (Additional file 1: Supplementary Fig. 5), broadly consistent with the previous MNase-seq results suggesting that nucleosome disruption is induced by DVNPs [58]. This disruption is, however, apparently not sufficient to dramatically reshape the accessibility landscape and represents a moderate quantitative rather than a major qualitative alteration.

### Inverse correlation between 5-hmU and chromatin accessibility

Next, we examined the distribution of 5-hmU and chromatin accessibility around other available genomic features. We noticed that in many cases (although this is not an exclusive association) 5-hmU is enriched around the boundaries of dinoTADs while ATAC-seq shows decreased accessibility in those same regions (Fig. 2A). We generalized this observation by evaluating the global ATAC-seq and 5-hmU distribution around dinoTAD boundaries and found that indeed ATAC-seq is globally depleted nearby these locations (Fig. 2B), while MeDIP-seq is enriched and 5-hmU chemical conversion rate is also elevated (Fig. 2C–D).

These observations extend globally to the whole genome, where ATAC-seq and 5-hmU levels display strong anticorrelation (Fig. 2E–F). This anti-correlation between chromatin accessibility and 5-hmU is specifically strong around dinoTAD boundaries while we do not observe a substantial inverse correlation between the two in the middle of dinoTAD domains (Fig. 2G). However, these observations are trends and not universal patterns, as a number of gene array boundaries do not show strong MeDIP enrichment and ATAC-seq depletion.

### Association of 5-hmU and chromatin accessibility with repetitive elements

**Fig. 3:**
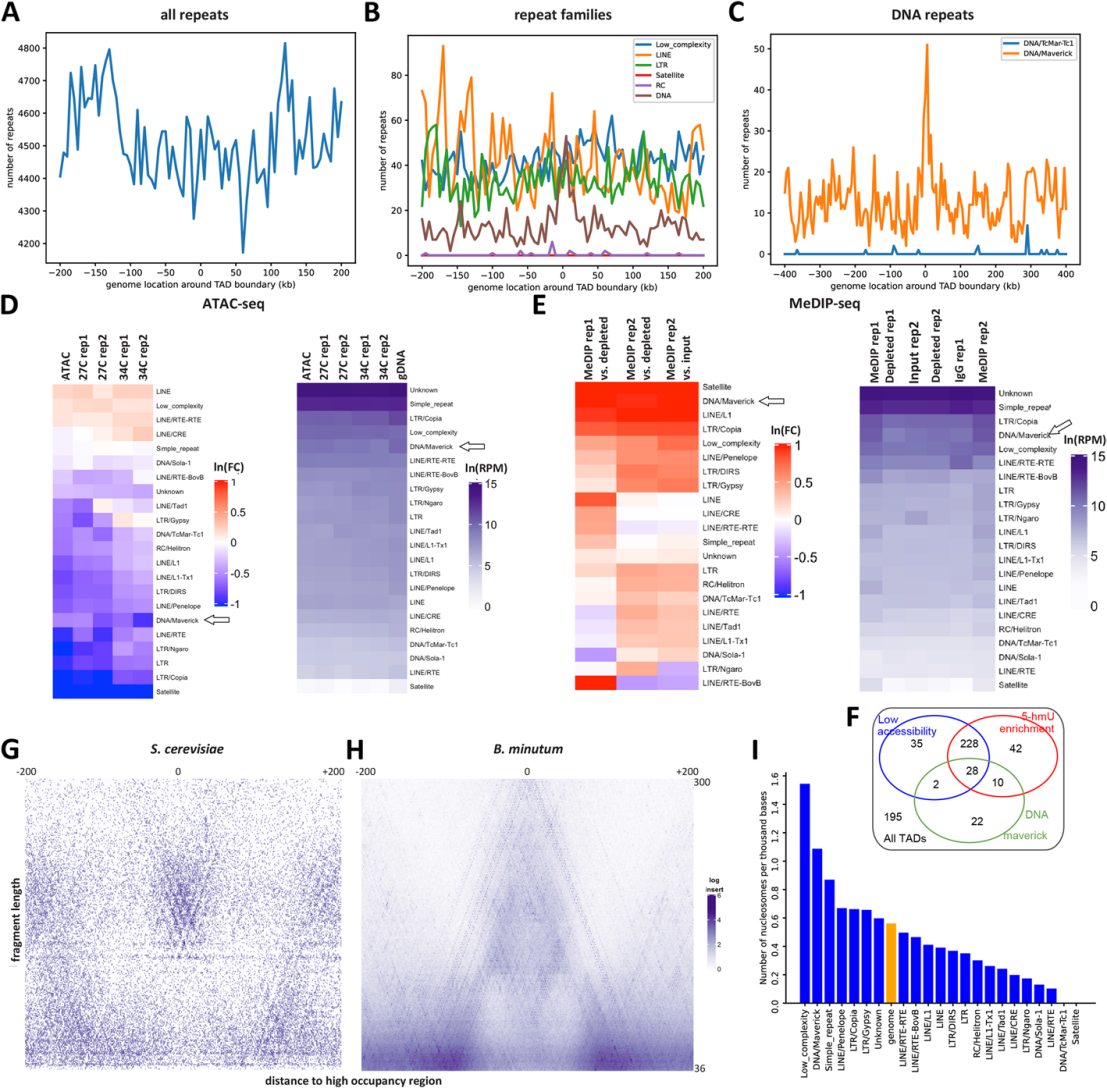
Association of 5-hmU and chromatin accessibility with repetitive elements in the *B. minutum* genome. **A**–**C** Distribution of all repeats, individual repeat families, and some DNA elements around dinoTAD boundaries. **D** ATAC-seq enrichment/depletion over repetitive elements. **E** MeDIP-seq enrichment/depletion over repetitive elements. **F** Overlap between dinoTAD boundaries, regions of low accessibility, regions of high 5-hmU, and DNA Maverick elements. **G**, **H** V-plot [68] around positioned nucleosomes in *S. cerevisiae* (for comparison) and de novo identified putative positioned nucleosomes in *B. minutum*. **I** Enrichment/depletion of positioned nucleosomes over repetitive elements

Because of the previously noted enrichment and depletion of multimapping reads in 5-hmU and ATAC libraries, respectively, we next aimed to identify which, if any, repetitive elements might be specifically associated with 5-hmU and/or ATAC. We first examined the distribution of annotated repetitive elements (see Methods for details) around dinoTAD boundaries (Fig. 3A–C), and found no specific preference at dinoTAD boundaries neither for repeats as a whole, nor for any specific repeat family, with one exception — Maverick DNA elements did exhibit strong enrichment around the edges of dinoTADs (Fig. 3C). Maverick elements are also known as Polintons, are typically 15–40 kbp in size, and often encode putative viral capsid proteins, suggesting that they might form virions under some conditions [62–66], a view supported by the large abundance of Polinton-like viruses reported in aquatic ecosystems [67]. Maverick does not account for all dinoTAD boundaries though — while a majority of TAD boundaries show 5-hmU enrichment, Maverick elements are found around only ∼11% of them (Fig. 3F).

Global analysis of ATAC-seq depletion/enrichment over repetitive elements (Fig. 3D) showed that most repeats are depleted for accessibility, with Copia LTR and Maverick DNA elements most highly abundant in the gDNA control relative to the ATAC-seq sample. The exceptions are CRE and RTE-RTE LINE elements. MeDIP-seq data reveals a generally inverse picture — most repeats are enriched in the MeDIP libraries, apart from some LINE elements (Fig. 3E), with Maverick/Polinton DNA repeats most strongly enriched for the 5-hmU modification.

These results point to increased protein occupancy and elevated 5-hmU levels over repetitive elements such as Maverick. We therefore asked whether we can specifically find nucleosomes corresponding to certain repeat classes. We utilized the nucleoATAC algorithm [69] to identify positioned nucleosomes genome-wide in the *B. minutum* genome (see the “ Methods” section; with the caveat that the nucleoATAC algorithm was designed to look for eukaryote-like nucleosomes and we do not know if this is still the case in dinoflagellates). We identified 30,107 low-resolution and 2,166 high-resolution putative positioned nucleosomes; these are overall not preferentially located to well-defined general genomic features such as dinoTAD boundaries (Additional file 1: Supplementary Fig. 6). V-plot analysis [68] of the fragment distribution around positioned nucleosomes revealed an A-shaped structure, with a peak in the 120–160 bp range (this fragment length is higher for the smaller set of highresolution nucleosomes; Additional file 1: Supplementary Fig. 6), flanked by very short fragments. This observation is distinct from what is observed in other eukaryotes such as yeast (Fig. 3G–H), where multiple nearby nucleosomes are visible. We interpret these structures as arising from a single-positioned protective feature, quite possibly a histone-based nucleosome, without other strongly positioned nearby nucleosomes. We note that these observations are not explainable by mappability biases (i.e., only a single nucleosome is observed because all adjacent sequences are not mappable), as we carried out this analysis while allowing for multimapping reads and the center point of the putative positioned nucleosomes is in fact slightly less uniquely mappable than the flanks (Additional file 1: Supplementary Fig. 7).

Strikingly, Maverick DNA elements are preferentially enriched for positioned nucleosomes, at ∼2 × the genomic average (Fig. 3I). This observation corroborates the depletion of the ATAC-seq signal observed over these elements.

## Discussion

In this study, we provide the first global maps of the distribution of the 5-hmU modification and chromatin accessibility in a dinoflagellate species (*B. minutum* in the Symbiodiniaceae clade). Our results point to the preferential enrichment for 5-hmU over certain classes of repetitive elements and also around the boundaries of the previously identified dinoTAD topologically associating domains that also coincide with the points of convergence of the long unidirectional arrays into which dinoflagellate genes are organized.

In contrast, chromatin accessibility is depleted in those same areas and is generally anti-correlated with high levels of 5-hmU. We do not observe strong accessibility peaks as seen in eukaryotes with conventional nucleosomal chromatin, nor do we see any preferential accessibility around transcription start sites, suggesting that most of the dinoflagellate genome is not protected by strings of nucleosomes and is generally uniformly physically accessible. We also do not see evidence for large-scale domains of preferential accessibility/protection; previously ATAC-see [70] was used to visualize through microscopy the accessible chromatin in the cells of the basal dinoflagellate *Hematodinium* [13], which suggested the existence of large-scale “open” and “closed” compartments, perhaps encompassing whole chromosomes. However, no ATAC sequencing data is available for *Hematodinium*, thus we do not know how these open/closed compartments translate into genome-wide sequencing profiles. It is possible that the open compartment is fully and uniformly open and the closed one is fully and uniformly closed, i.e., without distinct loci of open chromatin, resulting in a similar average genomewide profile to what we observe. Alternatively, chromatin structure in basal and core dinoflagellates (Symbiodiniaceae belonging to the latter) may differ substantially, as it is known that non-core dinoflagellates, such as the Syndiniales do not exhibit the typical dinokaryon structure and the complex of classic core dinoflagellate nuclear features emerges stepwise in the evolution of the group [26]. The application of single-molecule footprinting techniques to dinoflagellates should be able to resolve these possibilities in the future [60, 71], although at present it is hampered by the high sequencing coverage over the very large dinoflagellate genomes that is required. We do identify several thousand putative-positioned nucleosomes; however, these, if they are confirmed to be indeed histone-based nucleosomes, appear to be isolated and not parts of larger-order structures. An interesting trend that emerges is the association of elevated 5-hmU, decreased chromatin accessibility, and increased frequency of positioned nucleosomes over certain repetitive elements, in particular Maverick/Polinton DNA elements, which are also enriched over dinoTAD boundaries. This is, however, by no means an absolute rule as not all dinoTAD boundaries are associated with such features.

Nevertheless, it is tempting to draw parallels between these initial observations in dinoflagellates and what is known in much more detail in another major lineage of eukaryotes — the important mostly parasitic clade Kinetoplastida [72] (belonging to the larger Euglenozoa lineage). There are many parallels between the genomic organization of dinoflagellates and kinetoplastids [72] — although kinetoplastids have conventional nucleosomal chromatin, they too have lost transcriptional regulation as a primary mechanism for controlling gene expression. and their genes are also organized into long arrays [73–76], with mature mRNAs being the product of *trans*-splicing [77–81]. These properties are shared with other members of the larger Euglenozoa lineage that have been studied, such as *Euglena gracilis* [82]. Curiously, kinetoplastids are also the one other lineage where 5-hmU has also been observed in non-negligible quantities [83]. However, in kinetoplastids, 5-hmU appears to be simply a precursor to the synthesis of the larger modification *β*-D-Glucopyranosyloxymethyluracil, better known as base J [84–86], which does play a significant role in their genomes. Base J replaces about 1% of thymines and is predominantly found in repetitive DNA, especially in telomeric regions [87–89], but more importantly, it also demarcates the boundaries between gene arrays [90] and likely prevents transcriptional readthrough events [91, 92]. The free-living *Euglena* also has base J [82], and thus 5-hmU too, and it is likely that so do all members of the larger Euglenozoa grouping (comprising the kinetoplastids, euglenids, diplonemids and a few smaller clades).

As Base J is synthesized through 5-hmU as an intermediate onto thymines already incorporated into DNA, 5-hmU is thus also localized to the same regions of the genome in the cases where it has been measured (e.g., in kinetoplastids [54, 93]). It is therefore possible that 5-hmU in dinoflagellates may play an analogous role to base J in kinetoplastids, even though they have not evolved the further chemical elaboration needed that for base J synthesis.

However, such a speculation would still leave many unanswered questions. First, what is the mechanistic role of 5hmU? In our previous work in which dinoTAD structures were discovered [48], we showed them to depend on transcriptional activity and to disappear upon blocking transcription, i.e., they are most likely the product of extreme transcription-induced DNA supercoiling. At the same time, 5-hmU has been reported to increase the flexibility of the DNA double helix [44–47], which may suggest a possible role for 5-hmU in alleviating the supercoiling stress under which dinoflagellate genomes appear to exist, but the mechanistic details of such a link are currently unclear.

Second, why does 5-hmU vary so much between different dinoflagellate species — from 12 to 68% where it has been assayed — and where is it located in the genome in the extreme cases? The preferential localization to dinoTAD boundaries suggested from our work is consistent with genome-wide rates of 5-hmU on the lower end of this spectrum (which is also what the available data for other Symbiodiniaceae points to [41]), as array boundaries are fairly localized and encompass a minor fraction of the whole genome. However, the *B. minutum* genome is relatively small for a dinoflagellate — only on the order of 1 Gbp — while other species have much larger and more repeat-rich genomes, which might be related to higher overall 5-hmU levels given the preference of 5-hmU for repetitive elements that we observe. It is quite possible that the primary role of 5-hmU is in modulating the activity of repetitive elements, and its association with some dinoTAD boundaries is a consequence of the genomic distribution of those repeats, rather than indicative of a specific boundaryrelated function for 5-hmU. To better understand its properties and functions, it will be important to assay 5-hmU in a wide variety of dinoflagellate species with diverse genomic characteristics.

Similarly, it will also be vital to obtain very high-quality genome assemblies to work with. For example, in our current work, we have not been able to test whether 5-hmU is strongly associated with telomeres the way base J is in kinetoplastids, as the currently available *B. minutum* assembly is of too poor quality to allow such analysis.

Finally, what is the precise role of histones, DVNPs, and HLPs in dinoflagellate genomes? Here, we demonstrate decreased chromatin accessibility over certain regions in the genome as well as the existence of nucleosome-like structures, which suggests the presence of nucleosomes along the genome. However, mapping of histone/DVNP/HLP occupancy using chromatin immunoprecipitation (ChIP) will be needed to generate direct genome-wide profiles of the distribution of these proteins along the genome. Currently, this is precluded due to the extreme sequence divergence of dinoflagellate histone proteins [28], which makes existing anti-histone antibodies unreliable reagents for carrying out such experiments in dinoflagellates. Establishing the absolute levels of protection/occupancy in dinoflagellate genomes, through the application of methylation-based (especially long read-based) and enzymatic approaches [94] will also be highly valuable.

## Conclusions

We mapped for the first time the genomic 5-hmU and chromatin accessibility in a dinoflagellate genome. We found inverse correlation between the two, and association of 5-hmU with repetitive elements, and reduced chromatin accessibility over repeats. DNA repeats, especially Maverick elements are particularly enriched for 5-hmU and show reduced chromatin accessibility. Consistent with the general absence of histone-organized chromatin in dinoflagellates, the chromatin accessibility landscape is otherwise largely uniform. We observe a possible enrichment for 5-hmU and the repetitive element it is preferentially associated with around gene array boundaries, though this tentative conclusion will have to be generalized/confirmed on the basis of higher-quality genome assemblies in the future.

## Methods

### *B. minutum* cell culture

The clonal axenic *Symbiodinium*/*Breviolum minutum* strain SSB01 was used in all experiments. Stock cultures were grown as previously described [95, 96] in Daigo’s IMK medium for marine microalgae (Wako Pure Chemicals) supplemented with casein hydrolysate (IMK + Cas) at 27 °C at a light intensity of 10 µmol photons m^−2^ s^−1^ from Philips ALTO II 25-W bulbs on a 12-h-light:12-h-dark cycle. The medium was prepared in artificial seawater (ASW).

### Genomic DNA isolation

*B. minutum* genomic DNA was isolated as previously described [95]. Briefly, cells were centrifuged at 1000 g for 5 min, then resuspended in 500 µL 1 × Cell Lysis Buffer (prepared by mixing equal volumes of 2 × Cell Lysis Buffer — 2% SDS, 400 mM NaCl, 40 mM EDTA, 100 mM Tris–HCl, pH 8.0 — and H_2_O) and vortexed. The lysed cells were mixed with an equal 500 µL volume of phenol:chloroform:isoamyl alcohol (25:24:1), and mixed well by inverting a few times. The phases were centrifugation at 13,000 g for 5 min, then the top phase was transferred to a new tube and treated with 4 µL Ribonuclease A (20 mg/mL) by incubating for 30 min at 37 °C.

DNA was purified by adding an equal volume of phenol:chloroform:isoamyl alcohol (25:24:1), mixing well and centrifuging at 13,000 g for 5 min, then transferring the top layer to a new tube, to which phenol:chloroform:isoamyl alcohol (25:24:1) was added again, and the centrifugation and top phase isolation was repeated. Then 2.5 × volumes of 100% EtOH were added and the mixture was incubated on ice for 30 min or at − 20 °C overnight. The solution was then centrifuged at 13,000 g at room temperature for 20 min, the pellet was washed with 70% EtOH, dried on air, and resuspended in 50 µL H_2_O.

### ATAC-seq experiments

ATAC-seq experiments were performed following the omniATAC protocol [56].

Briefly, ∼100 K *B. minutum* cells were centrifuged at 1000 g, then resuspended in 500 µL 1 × PBS and centrifuged again. Cells were then resuspended in 50 µL ATAC-RSB-Lysis buffer (10 mM Tris–HCl pH 7.4, 10 mM NaCl, 3 mM MgCl_2_, 0.1% IGEPAL CA-630, 0.1% Tween-20, 0.01% Digitonin) and incubated on ice for 3 min. Subsequently, 1 mL ATAC-RSB-Wash buffer (10 mM Tris–HCl pH 7.4, 10 mM NaCl, 3 mM MgCl_2_, 0.1% Tween-20, 0.01% Digitonin) were added, the tubes were inverted several times, and nuclei were centrifuged at 500 g for 5 min at 4 °C.

Transposition was carried out by resuspending nuclei in a mix of 25 µL 2 × TD buffer (20 mM Tris–HCl pH 7.6, 10 mM MgCl_2_, 20% dimethyl formamide), 2.5 µL transposase (custom produced) and 22.5 µL nuclease-free H_2_O, and incubating at 37 °C for 30 min in a Thermomixer at 1000 RPM.

Transposed DNA was isolated using the MinElute PCR Purification Kit (Qiagen Cat# 28,004/28006), and PCR amplified as previously described [56]. Libraries were purified using the MinElute kit and then sequenced on an Illumina NextSeq 550 instrument as 2 × 36mers or as 2 × 75mers.

### ATAC-seq control experiments

Genomic DNA controls for ATAC-seq were generated by transposing purified gDNA. Briefly, 100 ng of gDNA were mixed with 2 µL Tn5, 25 µL 2 × TD buffer, and H_2_O for a total volume of 50 µL, then incubated at 55 °C for 5 min. The reaction was stopped by immediately proceeding with DNA isolation using the MinElute kit. Libraries were generated as described above for ATAC-seq.

### Genome assemblies

Datasets were processed against either the original *B. minutum* assembly [33] or against the Hi-C scaffolded assembly for *B. minutum* previously described [48], which is based on the original fragmented assembly for this species [33] and scaffolded into chromosome-level contigs using Hi-C data following established protocols [97].

### General analysis procedures

Browser tracks generation, fragment length estimation, and other analyses were carried out using custom-written Python scripts (https://github.com/georgimarinov/GeorgiScripts).

### Mappability track generation

Mappability tracks were generated as by tiling the whole genome with reads of length *RL* starting at each position. These reads were mapped back to the genome using the same settings used for processing real datasets. Average mappability over each position was calculated as the ratio *RC/RL* between its read coverage *RC* and the read length *RL*.

### ATAC-seq data processing

Demultipexed FASTQ files were mapped as 2 × 36mers using Bowtie [98] with the following settings: -v 2-k 2-m 1–best –strata-X 1000. Duplicate reads were removed using picard-tools (version 1.99). This mapping generated a set of uniquely mapping alignments only.

For the purpose of the analysis of multimappers, alignments were generated with unlimited alignment multiplicity with the following settings: -v 2-a–best–strata-X 1000.

Normalization of multimappers was performed using the previously described [99, 100] method of dividing each alignment by its read multiplicity, i.e.:

1 $${S}_{c,i}=\frac{\sum_{{R\in R}_{c,i}}\frac{1}{{NH}_{R}}}{\frac{\left|R\right|}{{10}^{6}}}$$

Where *S*_*c,i*_ is the signal score for position *i* on chromosome *c* (in RPM, or reads per million mapped reads units), |*R*| is the total number of mapped reads, |*R*_*c,i*_| is the number of reads covering position *i* on chromosome *c*, and *NH*_*R*_ is the number of locations in the genome a read maps to.

### ATAC-seq peak calling

Peak calling was carried out using MACS2 [101], with the gDNA library as a control, and with the following settings: -g 569,785,352-fBAMPE. Differentially accessible regions were identified using DESeq2 [102].

### Analysis of positioned nucleosomes

The analysis of positioned nucleosomes was carried out using NucleoATAC [69]. We used the low-resolution nucleosome calling program nucleoatac occ with default parameters that require ATAC-seq data and genomic windows of interest and return a list of nucleosome positions based on the distribution of ATAC-seq fragment lengths centered at these positions. Sliding windows of 1 kbp in steps of 500 bp were taken as inputs, and redundant nucleosome positions were eventually discarded. V-plots were made by aggregating unique-mapping ATAC-seq reads centered around the positioned nucleosomes and mapping the density of fragment sizes versus fragment center locations relative to the positioned nucleosomes as previously described [68, 69].

### MeDIP-seq experiments

To prepare inputs for MeDIP-seq experiments, gDNA was first sonicated using a Qsonica S-4000 with a 1/16″ tip for 3 min, with 10 s pulses at intensity 3.5, and 20 s rest between pulses. The IP procedure was adapted from the protocol for ChIP-seq as previously described [103].

For each reaction, 100 µL of Protein A Dynabeads (ThermoFisher Cat # 10002D) were washed 3 times with a 5 mg/mL BSA solution. Beads were then resuspended in 1 mL BSA solution and 5 µL of *α*-5-hmU antibody (Abcam Cat # ab19735) were added. Coupling of antibodies to beads was carried out overnight on a rotator at 4 °C. Beads were again washed 3 times with BSA solution and resuspended in 100 µL of BSA solution.

Sheared genomic DNA (∼1 µg 1:1 mix of *B. minutum* and *Homo sapiens*) was end-repaired and adapters were ligated to it following the procedure of the NEBNext Ultra II DNA Library Prep Kit for Illumina (NEB, E7645S), purified using AMPure XP beads and eluted in 50 µL of H_2_O, and then denatured at 98 °C for 10 min. DNA was then immediately placed on ice, resuspended in 850 µL RIPA buffer (1 × PBS, 1% IGEPAL, 0.5% Sodium Deoxycholate, 0.1% SDS, Roche Protease Inhibitor Cocktail) and added to the beads, then incubated overnight on a rotator at 4 °C.

Beads were washed 5 times with LiCl buffer (10 mM Tris–HCl pH 7.5, 500 mM LiCl, 1% NP-40/IGEPAL, 0.5% Sodium Deoxycholate) by incubating for 10 min at 4 °C on a rotator, then rinsed once with 1 × TE buffer. Beads were then resuspended in 200 µL IP Elution Buffer (1% SDS, 0.1 M NaHCO_3_) and incubated at 65 °C in a Thermomixer (Eppendorf) with interval mixing to dissociate antibodies. Beads were separated from the DNA solution by centrifugation, and DNA was purified using the MinElute kit.

Library generation was completed by carrying out PCR following the rest of the steps of the NEBNext Ultra II DNA Library Prep Kit protocol, using 15 cycles of amplification. Final libraries were purified using AMPure XP beads.

Several control libraries were prepared — “Input” from the gDNA that was used as input to the immunoprecipitation, “Depleted” from the supernatant from the first bead separation after the incubation of DNA with beads, and “IgG”, generated from a parallel immunoprecipitation reaction that used only Protein A beads (without a primary antibody).

### MeDIP-seq data processing

MeDIP-seq libraries processing was carried out in the same way as that of ATAC-seq datasets.

### 5-hmU chemical mapping experiments

Chemical mapping of 5-hmU as carried out following the previously described by Kawasaki et al. chemical conversion method [54] with some modifications.

Briefly, sheared genomic DNA was used as input, and end prep and adapter ligation were carried out using the NEBNext Ultra II DNA Library Prep Kit. After the ligation step, DNA was purified using AMPure XP beads and eluted in 50 µL of H_2_O. DNA denaturation was performed by adding NaOH to a final concentration of 0.05 M and incubating at 37 °C for 30 min. Oxidation was carried out by adding 2 µL of KRuO_4_ solution (15 mM in 0.05 M NaOH) for the “harsh oxidation” condition and 2 µL of KRuO_4_ solution (1.5 mM in 0.05 M NaOH) for the “mild oxidation condition”, then incubating for 30 min at room temperature. Oxidized DNA was purified using AMPure XP beads and extension was carried out by mixing 13.5 µL DNA, 1.6 µL 100 mM MgSO_4_, 2 µL NEB Index Primer, 2 µL 10 × ThermoPol Reaction Buffer (NEB), 0.5 µL 10 mM dNTP mix, and 0.4 µL Bst DNA Polymerase, Large Fragment (NEB), then incubating for 1 h at 37 °C. PCR amplification was carried out using the NEB Ultra DNA Library Prep Kit, with 12 cycles of PCR. Final libraries were purified using AMPure XP beads.

### Processing of 5-hmU chemical mapping datasets

The slamdunk package [104] (https://t-neumann.github.io/slamdunk/), which was originally developed for the analysis of SLAM-seq [105] datasets (the SLAM-seq protocol also generates T → C conversions) was adapted to estimate 5-hmU conversion levels.

First, the genome was tiled into 500-bp bins starting every 100 bp. Second, sequencing reads were trimmed of adaptors using Trim Galore, and used as input to slamdunk together with the genome tiling with the following settings:

–max-read-length75-59-n 1,000,000-m–skip-sam.

### Repeat annotation

Repeats were identified de novo from the scaffolded assembly using RepeatModeler-2.0.1 with default parameters. Repeat annotations were subsequently generated using RepeatMasker-4.1.1 [106] with RMBlast-2.10.0 as the sequence search engine.

### Analysis of ATAC-seq and MeDIP-seq data in repeat space

Sequencing datasets were analyzed in repeat space as previously described [100]. Briefly, reads were mapped to consensus repeat sequences with relaxed settings (-e 200 instead of—v 2) and with unlimited multimappers. Normalization of multimapping reads was carried out as above.

### Heterologous expression of DVNPs in yeast

The MS46 *S. cerevisiae* strain [107] was used for all experiments.

For the experiments in Additional file 1: Supplementary Fig. 4, each DVNP was expressed from a SIVu-WTC846::TetPr-DVNP3xNLS-linker-3PK construct that was integrated into a single copy at the URA locus of the MS46 stain. The WTC846::TetPr promoter is reported previously [108]. Cells were grown in YPD media at 30 °C overnight and expression was induced by the addition of 200 nM anhydrotetracycline. Cells were collected 5 h after induction. Untransformed MS46 cells were used as control.

For the experiments in Additional file 1: Supplementary Fig. 5, each DVNP was expressed from a multicopy pRS416-GAL1pr-DVNP3xHA-NLS plasmid, as first reported in by Irwin et al. [58], that was transformed into MS46. Cells were grown in synthetic media lacking uracil + 2% raffinose at 30 °C and cultured overnight before expression was induced by the addition of 2% galactose to the media. Cells were collected 7 h after induction. MS46 transformed with an empty pRS416-GAL1pr construct was used as control.

### Yeast SMF experiments

Yeast SMF experiments were carried out as previously described [71, 109–111].

A 1:1 mixture of *S. cerevisiae* cells expressing DVNPs and *Candida glabrata* cells (used as a control for normalization, as previously described [61]) amounting to a total of 2*.*5 × 10^8^ cells was used as input. Cells in log phase (OD_660_ ≤ 1*.*0) were first centrifuged at 13,000 rpm for 1 min, then washed with 100 µL Sorbitol Buffer(1.4 M Sorbitol, 40 mM HEPES–KOH pH 7.5, 0.5 mM MgCl_2_), and centrifuged again at 13,000 rpm for 1 min. Cells were then spheroplasted by resuspending in 200 µL Sorbitol Buffer with DTT added at a final concentration of 10 mM and 0.5 mg/mL 100 T Zymolase, followed by incubating for 5 min at 30 °C at 300 rpm in a Thermomixer. The pellet was centrifuged for 2 min at 5000 rpm, washed in 100 µL Sorbitol Buffer, and centrifuged again at 5000 rpm for 2 min.

Cells were then resuspended in 100 µL ice-cold Nuclei Lysis Buffer (10 mM Tris pH 7.4, 10 mM NaCl, 3 mM MgCl_2_, 0.1 mM EDTA, 0.5% NP-40) and incubated on ice for 10 min. Nuclei were then centrifuged at 5000 rpm for 5 min at 4 °C, resuspended in 100 µL cold Nuclei Wash Buffer (10 mM Tris pH 7.4, 10 mM NaCl, 3 mM MgCl_2_, 0.1 mM EDTA), and centrifuged again at 5000 rpm for 5 min at 4 °C. Finally, nuclei were resuspended in 100 µL M.CviPI Reaction Buffer (50 mM Tris–HCl pH 8.5, 50 mM NaCl, 10 mM DTT).

Nuclei were then first treated with M.CviPI (GpC methyltransferase) by adding 200 U of M.CviPI (NEB), SAM at 0.6 mM and sucrose at 300 mM, and incubating at 30 °C for 7.5 min. After this incubation, 128 pmol SAM and another 100 U of enzymes were added, and a further incubation at 30 °C for 7.5 min was carried out. Immediately after, M.SssI treatment (CpG methyltransferase) was followed, by adding 60 U of M.SssI (NEB), 128 pmol SAM, and MgCl_2_ at 10 mM and incubation at 30 °C for 7.5 min.

The reaction was stopped by adding an equal volume of Stop Buffer (20 mM Tris–HCl pH 8.5, 600 mM NaCl, 1% SDS, 10 mM EDTA).

HMW DNA was isolated using the MagAttract HMW DNA Kit (Qiagen; cat # 67,563) following the manufacturer’s instructions.

Enzymatically labeled DNA was then sheared on a Covaris E220 and converted into sequencing libraries following the EM-seq protocol, using the NEBNext Enzymatic Methyl-seq Kit (NEB, Cat # E7120L).

### Yeast SMF data processing

Adapters were trimmed from reads using Trimmomatic [112] (version 0.36). Trimmed reads were aligned against a combined *S. cerevisiae sacCer3* plus *Candida glabrata* C_glabrata_CBS138 genome index using bwa-meth with default settings. Duplicate reads were removed using picard-tools (version 1.99). Methylation calls were extracted using MethylDackel (https://github.com/dpryan79/MethylDackel). Additional analyses were carried out using custom-written Python scripts (https://github.com/georgimarinov/GeorgiScripts).

Chemically mapped nucleosome positions in *S. cerevisiae* were obtained from Brogaard et al. (2012) [113] as previously described [71].

### Yeast ATAC-seq experiments

Yeast ATAC-seq experiments were carried out as previously described [71, 111].

Briefly, ATAC-seq was carried out on the same nuclei isolated for SMF as described above (before resuspension in M.CviPI Reaction Buffer), by resuspending nuclei with 25 µL 2 × TD buffer (20 mM Tris–HCl pH 7.6, 10 mM MgCl_2_, 20% Dimethyl Formamide), 2.5 µL transposase (custom produced) and 22.5 µL nuclease-free H_2_O, and incubating at 37 °C for 30 min in a Thermomixer at 1000 RPM. Transposed DNA was isolated using the DNA Clean & Concentrator Kit (Zymo, cat # D4014) and PCR amplified as described before [56]. Libraries were then sequenced on an Illumina NextSeq instrument as 2 × 36mers or as 2 × 75mers.

### ATAC-seq data processing

FASTQ files were mapped against a combined *S. cerevisiae sacCer3* plus *Candida glabrata* C_glabrata_CBS138 genome index as 2 × 36mers using Bowtie [98] with the following settings: -v 2-k 2-m 1–best–strata. Duplicate reads were removed using picard-tools (version 1.99). Additional analysis was carried out as previously described [114].

### Supplementary Information

**Additional file 1: Supplementary Figure 1.** Representative genome browser view of ATAC-seq and gDNA control signal in the *B. minutum *genome. Note the presence of collapsed repeats visible in the gDNA track. **Supplementary Figure 2.** Representative genome browser view of ATAC-seq and gDNA control signal in the *B. minutum *genome. Note the presence of collapsed repeats visible in the gDNA track. **Supplementary Figure 3.** Relative degree of ATAC-seq enrichment in *B. minutum *versus a representative mammalian genome sample. Shown is the *log*_2_(fold change) ratio of ATAC-seq signal versus a negative control for a representative human ATAC-seq sample (K562 cell line from the ENCODE Project Consortium^58^; dataset ID ENCFF512VEZ was used for ATAC and dataset ID ENCFF285UKJ — a whole genome bisulfite sequencing library — as a negative control, over peaks from dataset ID ENCFF695IGF). A separately sequenced gDNA control was generated for the *B. minutum *ATAC. **Supplementary Figure 4.** Effects of exogenous expression dinoflagellate DVNPs on chromatin accessibility in the yeast *S. cerevisiae*. (A-B) ATAC-seq profiles of *S. cerevisiae *expressing *B. minutum *DVNP symbB.v1.2.006931 and *Hematodinium *sp. DVNP.12 and control samples. (C) SMF profiles (corrected using average SMF methylation from the *Candida *internal control) over *S. cerevisiae *TSSs in *S. cerevisiae *expressing *B. minutum *DVNP symbB.v1.2.006931 and *Hematodinium *sp. DVNP.12 and control samples. (D) SMF profiles (corrected using average SMF methylation from the *Candida *internal control) over positioned *S. cerevisiae *nucleosomes in *S. cerevisiae *expressing *B. minutum *DVNP symbB.v1.2.006931 and *Hematodinium *sp. DVNP.12 and control samples. **Supplementary Figure 5.** Effects of exogenous expression of dinoflagellate DVNPs on chromatin accessibility in the yeast *S. cerevisiae*. ATAC-seq profiles of *S. cerevisiae *expressing *Hematodinium *sp. DVNP.5 (from Irwin et al. 2018^59^) and a vehicle control, as well as additional replicates for *B. minutum *DVNP symbB.v1.2.006931 and *Hematodinium *sp. DVNP.12 and control samples. “OFF” and “ON” refer to cells in which the expression of *Hematodinium *sp. DVNP.5 is induced or not. **Supplementary Figure 6.** Positioned nucleosomes as a whole are not strongly enriched around dinoTAD boundaries. **Supplementary Figure 7.** Properties of putative positioned nucleosomes in the *B. minutum *genome. (A) V-plot of low-resolution positioned nucleosomes (*n*=30,107) (B) V-plot of low-resolution positioned nucleosomes with minimum occupancy cutoff of 0.8 (*n*=2,166) (C) Fragment distribution over low-resolution positioned nucleosomes (D) Fragment distribution over high-resolution positioned nucleosomes with minimum occupancy cutoff of 0.8 (E) Average mappability (for reads of length 75 bp) over positioned nucleosomes.

**Additional file 2.** Review history.

## Data Availability

Data associated with this manuscript have been submitted to GEO under accession number GSE241969 [115]. The data processing and visualization code used is available under the MIT license on GitHub [116] and Zenodo [117].
