# Supplementary material for: Genome-wide distribution of 5-hydroxymethyluracil and chromatin accessibility in the Breviolum minutum genome
Source: Genome Biol. 2024 May 6;25:115. doi: 10.1186/s13059-024-03261-3 (PMC11071213; doi:10.1186/s13059-024-03261-3)
Supplement: Supplementary file 1 — Additional file 1: Supplementary Figure 1. Representative genome browser view of ATAC-seq and gDNA control signal in the B. minutum genome. Note the presence of collapsed repeats visible in the gDNA track. Supplementary Figure 2. Representative genome browser view of ATAC-seq and gDNA control signal in the B. minutum genome. Note the presence of collapsed repeats visible in the gDNA track. Supplementary Figure 3. Relative degree of ATAC-seq enrichment in B. minutum versus a representative mammalian genome sample. Shown is the log2(fold change) ratio of ATAC-seq signal versus a negative control for a representative human ATAC-seq sample (K562 cell line from the ENCODE Project Consortium58; dataset ID ENCFF512VEZ was used for ATAC and dataset ID ENCFF285UKJ — a whole genome bisulfite sequencing library — as a negative control, over peaks from dataset ID ENCFF695IGF). A separately sequenced gDNA control was generated for the B. minutum ATAC. Supplementary Figure 4. Effects of exogenous expression dinoflagellate DVNPs on chromatin accessibility in the yeast S. cerevisiae. (A-B) ATAC-seq profiles of S. cerevisiae expressing B. minutum DVNP symbB.v1.2.006931 and Hematodinium sp. DVNP.12 and control samples. (C) SMF profiles (corrected using average SMF methylation from the Candida internal control) over S. cerevisiae TSSs in S. cerevisiae expressing B. minutum DVNP symbB.v1.2.006931 and Hematodinium sp. DVNP.12 and control samples. (D) SMF profiles (corrected using average SMF methylation from the Candida internal control) over positioned S. cerevisiae nucleosomes in S. cerevisiae expressing B. minutum DVNP symbB.v1.2.006931 and Hematodinium sp. DVNP.12 and control samples. Supplementary Figure 5. Effects of exogenous expression of dinoflagellate DVNPs on chromatin accessibility in the yeast S. cerevisiae. ATAC-seq profiles of S. cerevisiae expressing Hematodinium sp. DVNP.5 (from Irwin et al. 201859) and a vehicle control, as well as additional replicates for B. minutum [file 13059_2024_3261_MOESM1_ESM.pdf]

Supplementary Materials

Supplementary Figures

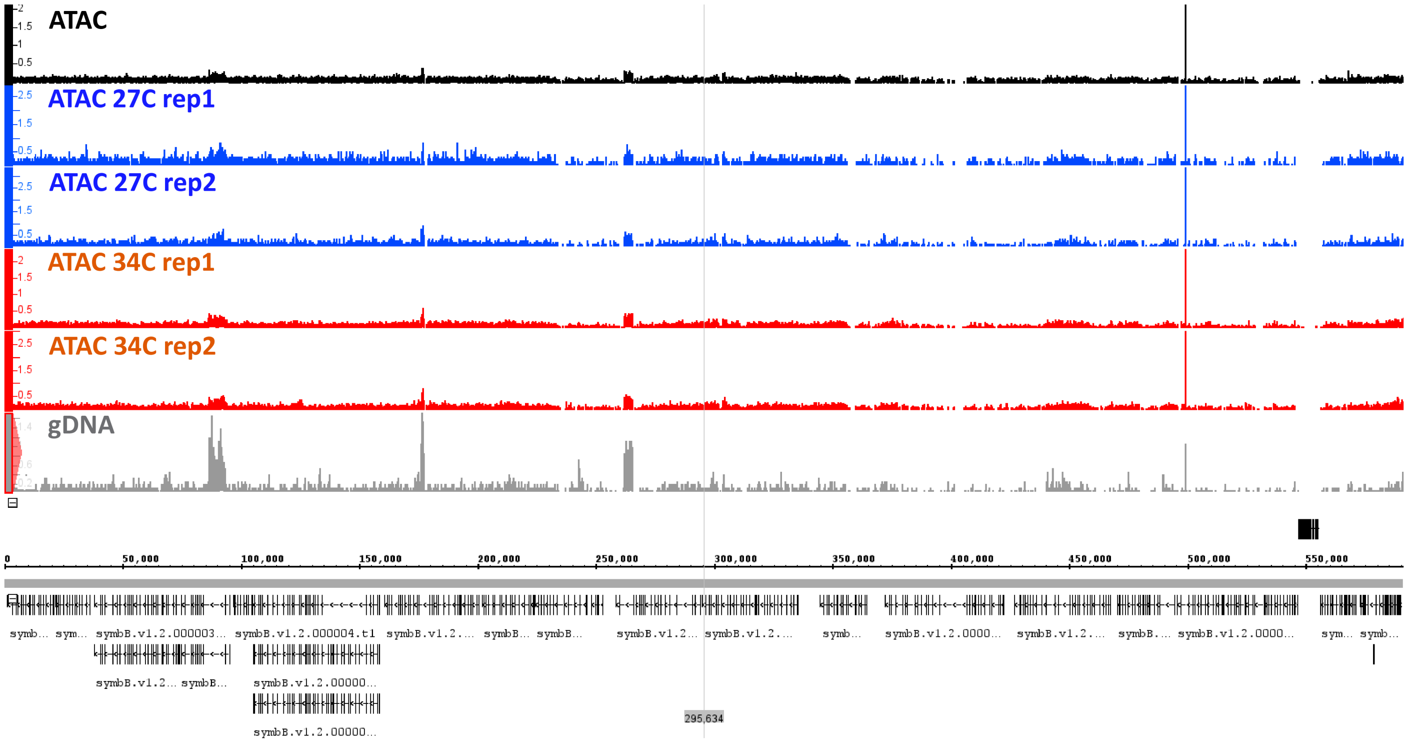

Supplementary Figure 1: Representative genome browser view of ATAC-seq and gDNA control signal in the *B. minutum* genome. Note the presence of collapsed repeats visible in the gDNA track.

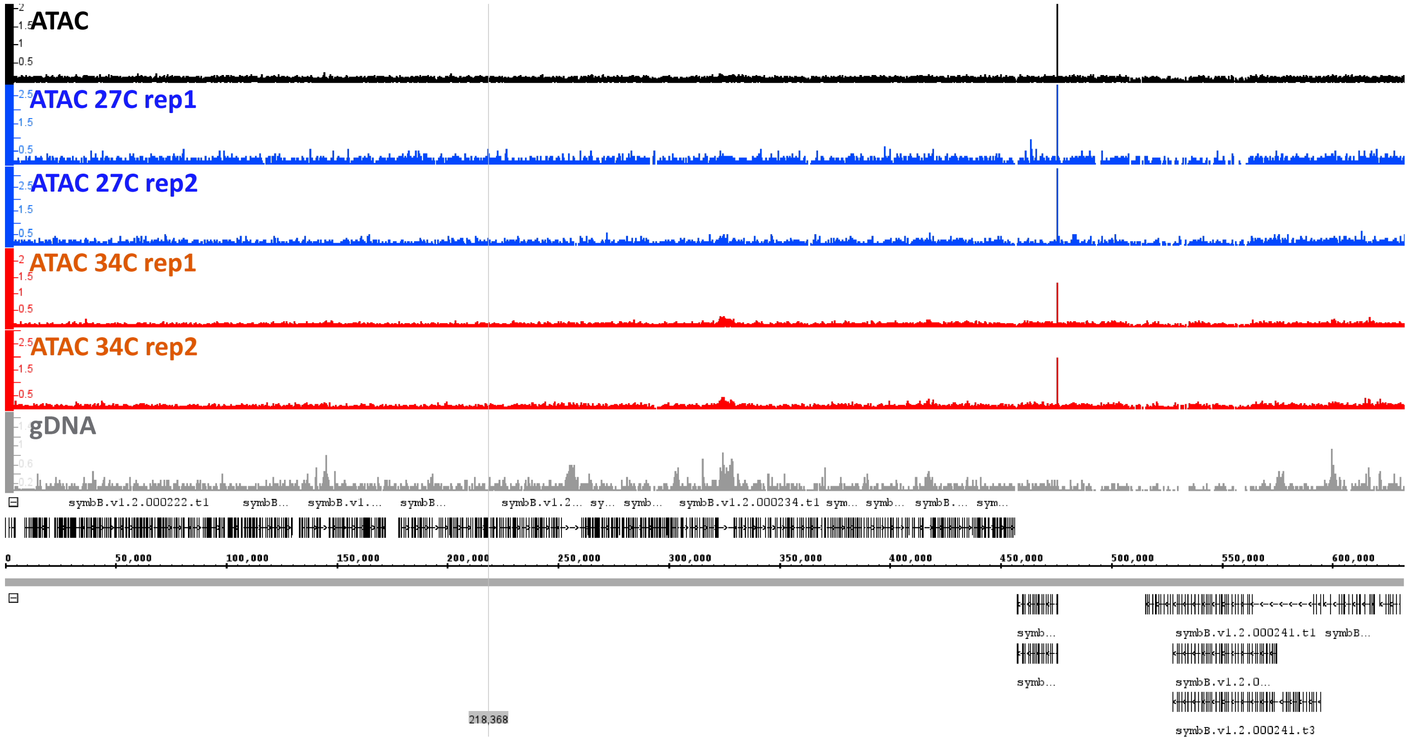

Supplementary Figure 2: Representative genome browser view of ATAC-seq and gDNA control signal in the *B. minutum* genome. Note the presence of collapsed repeats visible in the gDNA track.

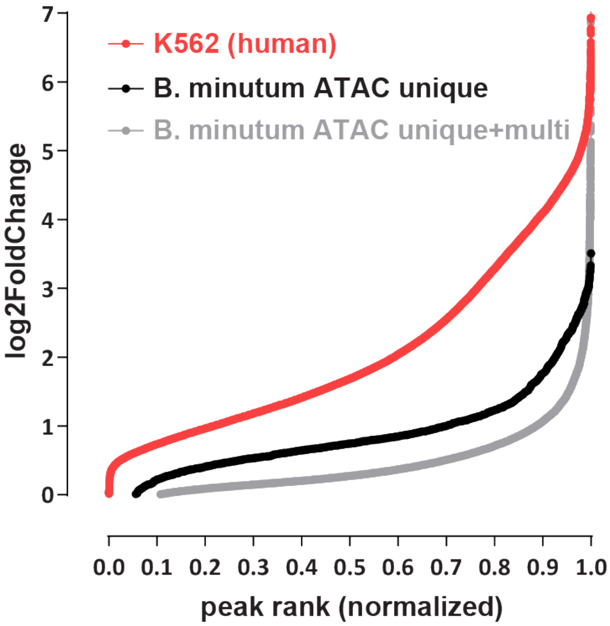

Supplementary Figure 3: Relative degree of ATAC-seq enrichment in *B. minutum* versus a representative mammalian genome sample. Shown is the log_2(fold change) ratio of ATAC-seq signal versus a negative control for a representative human ATAC-seq sample (K562 cell line from the ENCODE Project Consortium[58]; dataset ID ENCFF512VEZ was used for ATAC and dataset ID ENCFF285UKJ – a whole genome bisulfite sequencing library – as a negative control, over peaks from dataset ID ENCFF695IGF). A separately sequenced gDNA control was generated for the *B. minutum* ATAC.

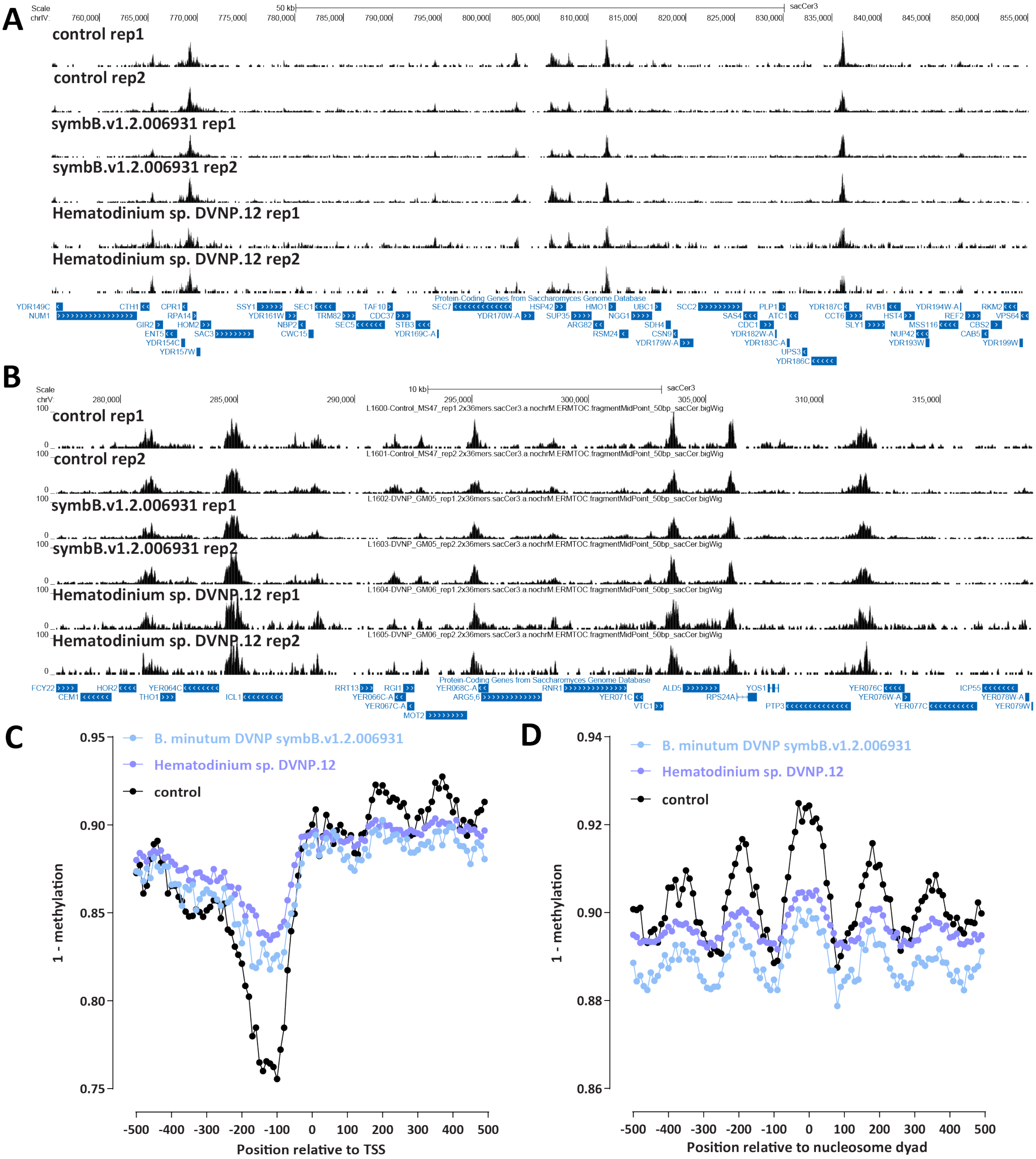

Supplementary Figure 4: Effects of exogenous expression dinoflagellate DVNPs on chromatin accessibility in the yeast *S. cerevisiae*. (A-B) ATAC-seq profiles of *S. cerevisiae* expressing *B. minutum* DVNP symbB.v1.2.006931 and *Hematodinium* sp. DVNP.12 and control samples. (C) SMF profiles (corrected using average SMF methylation from the *Candida* internal control) over *S. cerevisiae* TSSs in *S. cerevisiae* expressing *B. minutum* DVNP symbB.v1.2.006931 and *Hematodinium* sp. DVNP.12 and control samples. (D) SMF profiles (corrected using average SMF methylation from the *Candida* internal control) over positioned *S. cerevisiae* nucleosomes in *S. cerevisiae* expressing *B. minutum* DVNP symbB.v1.2.006931 and *Hematodinium* sp. DVNP.12 and control samples.

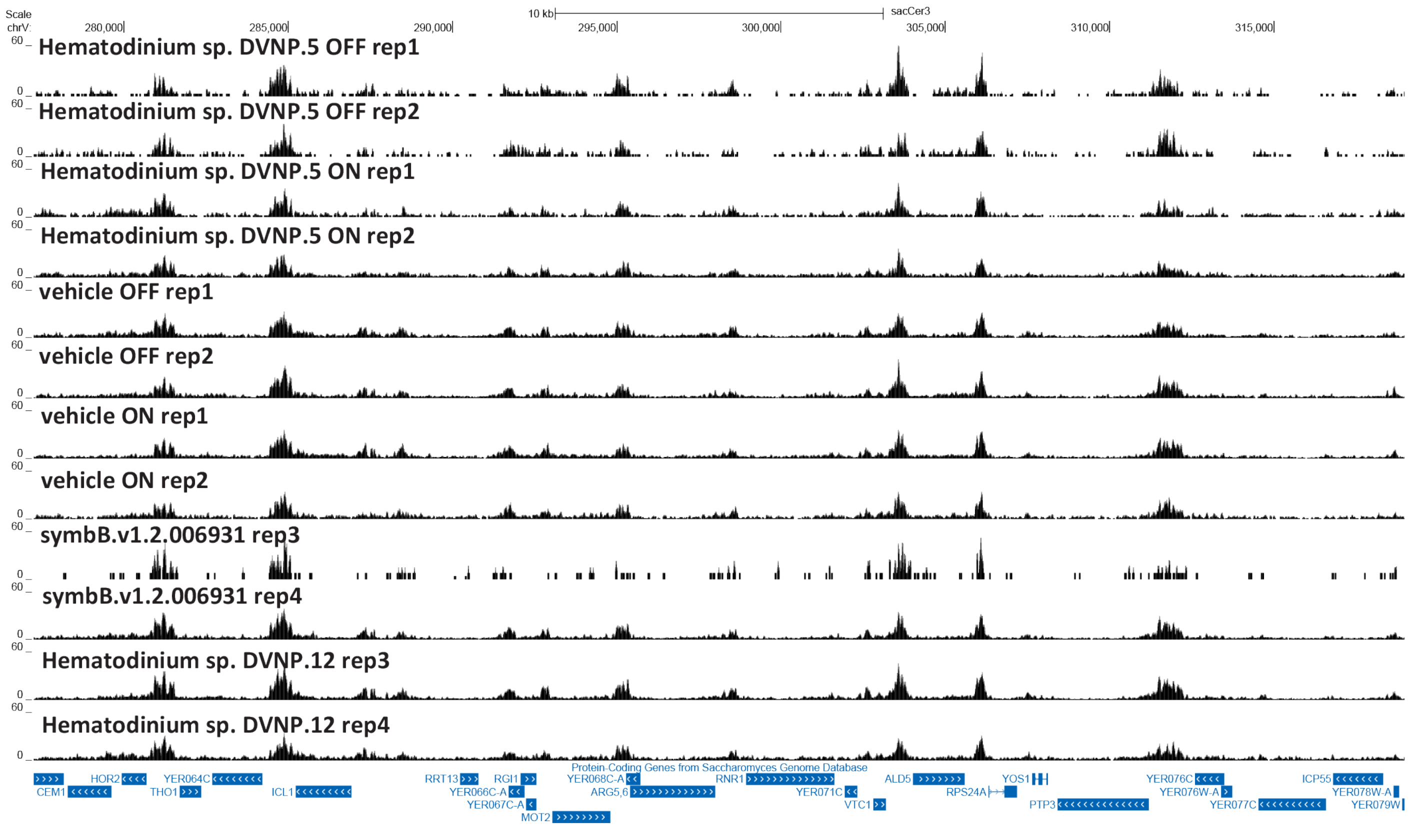

Supplementary Figure 5: Effects of exogenous expression of dinoflagellate DVNPs on chromatin accessibility in the yeast *S. cerevisiae*. ATAC-seq profiles of *S. cerevisiae* expressing *Hematodinium* sp. DVNP.5 (from Irwin et al. 2018[59]) and a vehicle control, as well as additional replicates for *B. minutum* DVNP symbB.v1.2.006931 and *Hematodinium* sp. DVNP.12 and control samples. "OFF" and "ON" refer to cells in which the expression of *Hematodinium* sp. DVNP.5 is induced or not.

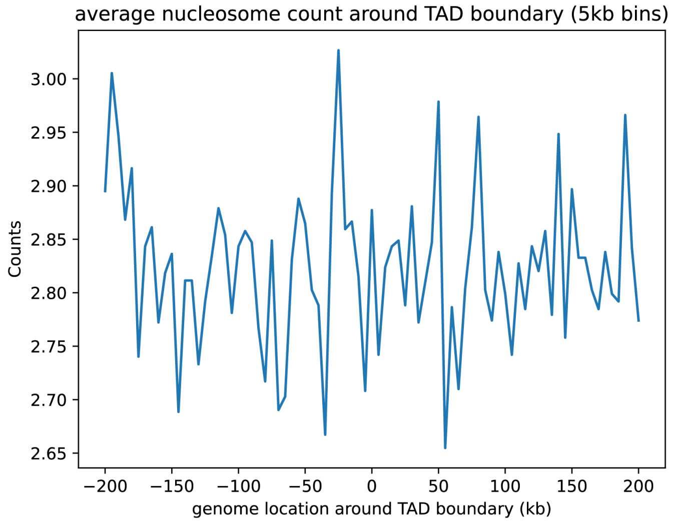

Supplementary Figure 6: Positioned nucleosomes as a whole are not strongly enriched around dinoTAD boundaries.

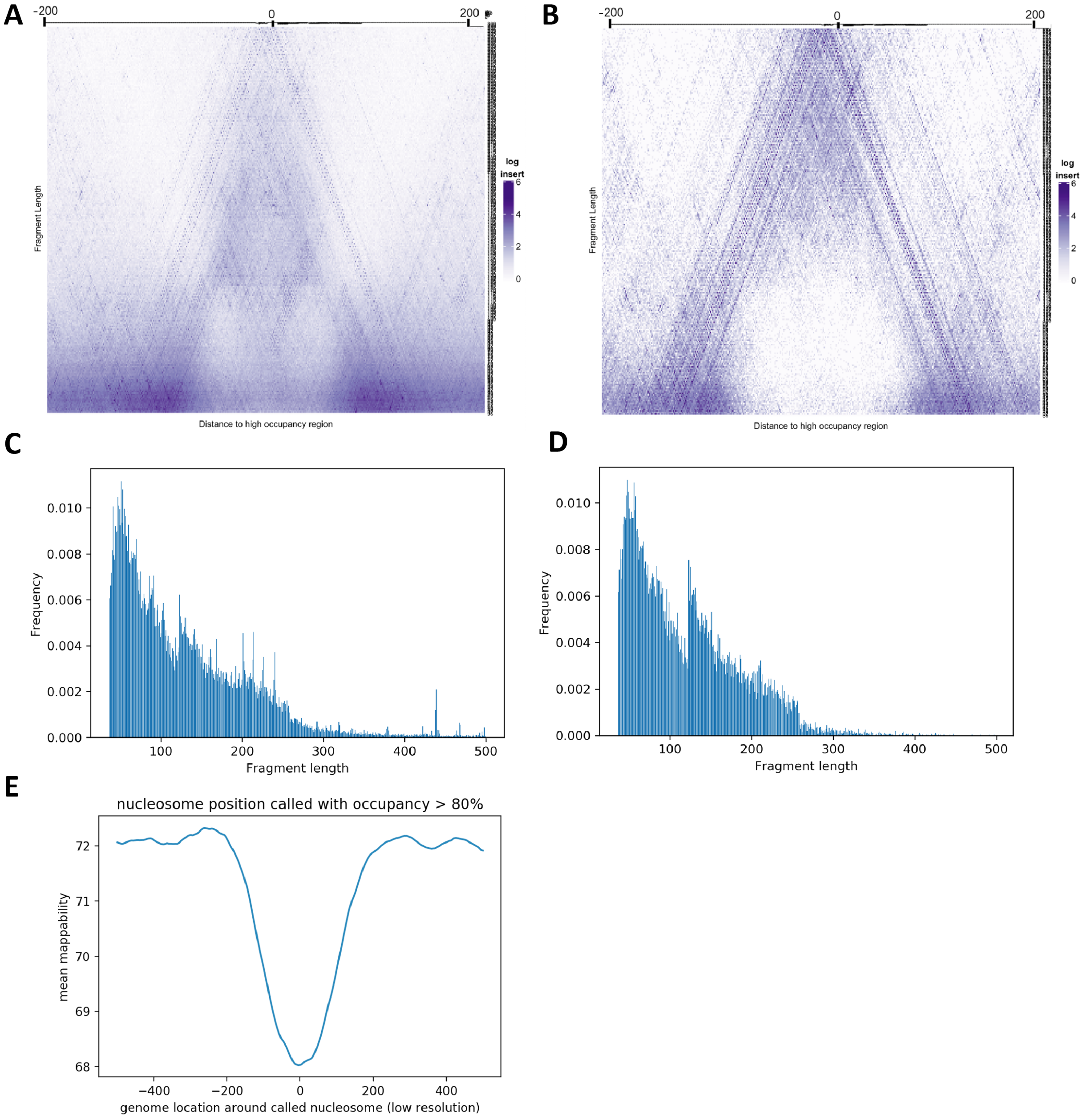

Supplementary Figure 7: Properties of putative positioned nucleosomes in the *B. minutum* genome. (A) V-plot of low-resolution positioned nucleosomes (n=30,107) (B) V-plot of low-resolution positioned nucleosomes with minimum occupancy cutoff of 0.8 (n=2,166) (C) Fragment distribution over low-resolution positioned nucleosomes (D) Fragment distribution over high-resolution positioned nucleosomes with minimum occupancy cutoff of 0.8 (E) Average mappability (for reads of length 75 bp) over positioned nucleosomes.
