# Supplementary material for: Genome-wide distribution of 5-hydroxymethyluracil and chromatin accessibility in the Breviolum minutum genome
Source: Genome Biol. 2024 May 6;25:115. doi: 10.1186/s13059-024-03261-3 (PMC11071213; doi:10.1186/s13059-024-03261-3)
Supplement: Supplementary file 2 — Additional file 2. Review history. [file 13059_2024_3261_MOESM2_ESM.docx]

Review History

**First round of review**

**Reviewer 1**

**Were you able to assess all statistics in the manuscript, including the appropriateness of statistical tests used?**

Yes, and I have assessed the statistics in my report.

**Comments to author:**

Dinoflagellates are photosynthetic Eukaryotes that play an important ecological role in marine ecosystems by providing endosymbiotic and metabolic support to coral reefs. Dinoflagellates are eukaryotes with unusual genome compaction strategies that include histone-based chromatinization in addition to DVNPs and HLPs that result in permanently condensed liquid crystalline chromosomes. It is unclear how DVNPs, HLPs, and these divergent histones function in dinoflagellate nuclei. It is known that 5hmU is rare in most eukaryotes but highly abundant in dinoflagellates. The chromatin landscape nor the function of 5hmU are known in the dinoflagellate, B. minutum. It is known that chromosomes adopt three-dimensional distinct domain topology, and this study levies previous Hi-C data to correlate 5hmU and chromatin accessibility.

The manuscript entitled "Genome-wide distribution of 5-hydroxymethyluracil and chromatin accessibility in the Breviolum minutum genome" by Marinov at al. is the first study to report a global map of the distribution of DNA 5hmU and chromatin accessibility in the dinoflagellate species, B. minutum, and adds credence to previous studies that demonstrate 5hmU is abundant in this species. Perhaps surprisingly, the current research did not detect a sequence logos that describes the context of 5hmU that previous studies have found in related species. Instead, the current study reports that 5hmU is enriched in repetitive regions and at topological associated domain (TAD) boundaries but depleted in chromatin accessible regions. Repeat regions tend to be less accessible to ATAC-seq, and there is an anti-correlation between 5hmU and chromatin accessibility. The B. minutum genome is largely accessible, and there does not appear to be any correlation between putative nucleosome positions and TAD boundaries. But, Maverick DNA elements are enriched near TAD boundaries and for putative nucleosome signals. This study reports that many 5hmU reads mapped within Maverick DNA elements, but this is not an exclusive relationship, as most 5hmU reads and residues are found outside of Maverick DNA elements. The totality of the data/interpretations lead to a model wherein 5hmU may play a role in stabilizing dinoTAD boundaries and promoting chromatinization, but this conclusion is based on descriptive and correlative data rather than direct evidence.

The manuscript is received and reviewed with mixed feelings: while many figures could be improved, the methodology and statistical analyses appear to be done quite well. While the analyses are complete, the impactful nature of the results obtained is not sufficiently described or clear…thus, will the community of readers gain much from the descriptive nature of the potential role of 5hmU? This study appears unwilling or limited in its ability to make conclusive statements concerning 5hmU, TADs, and chromatinization and thus it is difficult to determine its overall impact. In general, the results presented are novel, but the implications/relevance of the findings are not well described. It is likely that a revised and conclusion-focused revision could lead to large improvements.

Comments

* Figure 2: missing panel E; panels B, F, and G do not appear to be referenced in the text. The text does not describe panels F-H at all. The colors and shapes of the circles mean something (surely) but are not described. Mild/harsh oxidation conditions are not explained in sufficient detail for the meaning to be clear. Care should be taken to review each figure for clarity and complete descriptions.

* Figure 3D could be improved by aligning the x-axis labels.

* Figure 3D figure legend, "S. cerevisiae" should be italicized.

* The statement "These results point to increased protein occupancy and…" should be justified and supported with data. Is there any direct evidence of increase protein occupancy? Could such data be generated?

* grammar - line 3 column 1, "an eukaryote" → "a eukaryote"

* grammar Lines 28 column 1, "In order the map…" → " In order to map…"

* Figure 1D. The logos sequence presentation could be improved for clarity. Consider making the Red line at position 6 a "U" or a "T".

* Supplementary Figure 1 and 2, why are there several strong ATAC-seq peaks in the gDNA control? Are these the collapsed repeats? It would be ideal if the collapsed repeat regions were clearly labeled.

* Figure 1G is perplexing. It is difficult to determine/understand that the gDNA control was replicated at 27 and 34 degrees C, but it is unclear what the ATAC curve is referring to, or why the line is black but the text is red. In Figure 1H, it is similarly unclear what the "ATAC" control is in bar 1.

* Typo -- line 46 column 1, "normal temperature (34 C) condition…" → "normal temperature (27 C) condition…"

* Supp. Figure 3, What is peak range? It is currently unclear what the x-axis reports.

* "Hematodinium sp. DVNP.5 had a negative effect on growth, as previously reported, but much more strongly so than the other two." Should there be a data shown here to support this statement?

* Typo - line 58 column 2, "more strongly so than the other two"

* Supp Figure 4: it is unclear what the difference is between panel A and B.

* The manuscript would benefit from a short description of the SFM methodology in the text. Supp. Figure 4 panel C and D mention methylation on the y-axis, and but is not sufficiently explained to allow interpretation.

* Typo - line 61-62 column 2, "ATAC-see" → "ATAC-seq"

**Reviewer 2**

**Were you able to assess all statistics in the manuscript, including the appropriateness of statistical tests used?**

No, I do not feel adequately qualified to assess the statistics.

**Comments to author:**

This manuscript reports a series of experiments aimed at characterizing aspects of the chromatin of the symbiotic dinoflagellate Breviotum minutum. In some ways this is a continuation of the effort to shed light on the long mystery of dinoflagellate chromatin, started by the authors recently with the first genome-wide analysis of chromatin conformation in a dinoflagellate. The present study touches on a key issue, which is the presence of the modified base 5-hmU, which substitutes Thymidine and which function, or roles have remained an open question for decades. The authors use a ChIP-seq approach to determine the distribution of 5-hmU (and an additional, chemical modification method) combined with genome wide ATAC-seq and leveraging the existing annotated genome reference, they analyze the relationships between 5-hmU enrichment with chromatin accessibility, TAD boundaries, unidirectional gene arrays, and transposable elements.

This study is important and welcomed as it provides important clues to start putting together the long standing puzzle of dinoflagellate chromatin architecture. However, as with any "first" that intends to show a path forward, addressing potential flaws and shortcoming is critical to sustain the credibility of the approaches. Below I list comments, questions and suggestions to improve the manuscript before its publication.

First and foremost, this study will arouse the interest of a lot of people that are not familiar with the latest advances in genomics. For them, it will be hard to follow the arguments without a lot of back and forth between the paper, references and other sources. I strongly suggest that a schematic figure with the main methods employed (mainly MeDIP-Seq, the mapping of chemically converted 5-hmU and ATACseq), be included to facilitate understanding by those who have followed the topic for decades but are not on top of the developments in the field.

General comment on figures: the captions are not useful to understand and interpret the figures. Readers should not be compelled to read the methods and even references to be able to get the main idea out of a figure. Prime example (but not the only one): figure 1D.

I think that to understand figs. 1A, B, H and F a schematic diagram of the procedure and rationale is required.

Pg. 3, col. 2 lines 33-44: what is the interpretation? Introduce for the readers the idea under multimapping reads.

Pg. 4, col 1 line 46: It says 34C both times. This analysis could be more interesting than what's portrayed by the authors. This result shows potentially hundreds of regions differentially accessible between the two temperature regimes. I am curious if there is anything interesting to say there.

Repair the labelling scheme of Fig. 2. Panel letters are messed up.

The experiments of heterologous expression of DVNP in yeast appears as an odd addition to the paper. I don't have a strong objection as there are some interesting tidbits from these experiments that add up and complement the story. However, cell growth data for transformed cultured expressing DVNP is missing.

Perhaps the answer to this is found in the references cited, but I'd like to see a short explanation for why a seemingly generalized oxidation reaction would only result in the conversion of 5-hmU into Cytosines without further effects on other bases (modified or otherwise).

I found the data in supplementary figure 6 too important to be buried there. I suggest make a table with all the numbers and including it in the manuscript. A table (matrix perhaps) that shows not only the numbers for the individual sections in the Venn diagram but also the sums (e.g., all 'low accessibility', all '5-hmU enrichment' and the grand total.

Other questions that I have which I don't think are responded:

How many TADs are there, and how many genes they include (mean and range)?

How reliable is the analysis of positioned nucleosomes (figs S7, 8)? I'm skeptical this has a real value, but I'm open to be convinced otherwise, and in that case, I'd like to hear some more about these nucleosomes, like where they are, how few or many they are compared to what's found in a similarly sized, "normal" eukaryotic genome.

**Reviewer 3**

**Were you able to assess all statistics in the manuscript, including the appropriateness of statistical tests used?**

There are no statistics in the manuscript.

**Comments to author:**

The manuscript "Genome-wide distribution of 5-hydroxymethyluracil and chromatin accessibility in the Breviolum minutum genome" maps the distribution of the unusual 5 hmU modification and the chromatin accessibility in the genome of the dinoflagellate B. minutum. The authors find that the 5-hmU modification is enriched over certain types of repetitive elements and also coincides with the boundaries between convergent gene arrays. The modification is also correlated with chromatin inaccessibility. The authors apply an impressive range of methods to study genome modification and organization of this dinoflagellate and discuss their findings briefly in the framework of other dinoflagellates and the only other group of eukaryotes known to possess higher quantities of the 5-hmU modification, the Kinetoplastida.

The analyses are comprehensive and sound and have resulted in very interesting findings. However, the manuscript would greatly benefit from a more detailed explanation of these findings (for non-specialists in these types of analyses and non-specialists in protists), a more stream-lined introduction and from an, in parts, more comprehensive discussion to make it more accessible and informative for a maximum range of readers.

Major comments (in order of appearance in the manuscript):

Pages 1-2 (Introduction): the introduction is in parts very detailed but it is often hard to understand why certain information is provided, how it is related to the focus of this work. E.g. what is the importance of the paragraph on 5mC methylation for this manuscript and how is this type of hypermethylation in the investigated Symbiodiniaceae relevant? (page 2, left column, lines 57-62).

There is a long, quite confusing paragraph about Kinetoplastida (that are only later introduced to belong to Euglenozoa) on that same page, suggesting this group will play an important role in the manuscript, however they are only mentioned briefly in the discussion.

Overall, the single paragraphs of the Introduction seem sometimes unrelated to each other and the whole introduction section would greatly benefit if they were better thematically connected - to each other and to the rest of the manuscript.

Page 2, right column, line 40: The authors describe the Kinetoplastida as "parasitic clade" which is not correct, as they contain several free-living genera that are common in various environmental habitats (e.g. bodonids).

Page 3, left column, lines 60-62: How does 5mC modification in mammals relate to the modification analyzed here? See also comment about the Introduction above.

Page 3, right column, lines 10-14: the authors state that the method used does not allow for estimation of absolute 5-hmU levels. What is the advantage of the method used here over the method(s) used to analyze the other dinoflagellates for which 5-hmU estimates are available?

Page 3, right column, 3rd paragraph: the authors state that the ATAC-seq chromatin accessibility analysis resulted in a peak at short fragment length of ~60 bp. Can the authors expand/speculate on this, why 60 bp? and can they provide the expected fragment lengths for nucleosomal chromatin for comparison (only the length for the subnucleosomal peak is given)?

Page 7, second paragraph: this two sentences seem contradictory: "We first examined the distribution of annotated repetitive elements (see Methods for details) around dinoTAD boundaries (Figure 3A-C), and found no specific preference at dinoTAD boundaries neither for repeats as a whole, nor for any specific repeat family. However, Maverick DNA elements did exhibit strong enrichment around the edges of dinoTADs." Please specify the intended meaning of these sentences. As it is, they are a bit confusing.

Discussion, page 8, right column, first paragraph: how do the authors explain the low (and similar to Symbiodinium microadriaticum) levels of 5-hmU in Prorocentrum (Exuviaella = basionym) cassubica? In that context, it would be also helpful for the readers to know that of all the dinoflagellates listed in the Intro as having been analyzed for 5-hmU, only S. minutum belongs to the Symbiodiniaceae, the rest are free-living species with genomes up to two magnitudes higher. This fact should be mentioned to put the 5-hmU numbers into context and maybe also to discuss whether genome organization in the symbiotic species and free-living species (with these massive genomes) can be expected to be the same or whether the differences may not just be in scale. Related also to the comment above - how comparable are those numbers for 5-hmU levels across all those different studies?

Minor comments:

Page 1, Intro, first paragraph: mention somewhere that dinoflagellates are unicellular eukaryotes. Readers unfamiliar with this lineage may not know.

Page 3, right column, line 46: which genome assembly was inspected in the genome browser? Two different assemblies are listed in the methods.

Page 3, right column, line 16: typo "B. minitum"

Page 4, right column, last sentence: it is a bit confusing why C. glabrata cells were used as control without reading the methods first.

Page 8, left column, first paragraph: maybe they authors can define in more detail what "fully open/closed" in this sentence means: "It is possible that the open compartment is fully open and the closed one is fully closed"

**Authors Response**

**Point-by-point responses to the reviewers’ comments:**

**Reviewer 1**

Dinoflagellates are photosynthetic Eukaryotes that play an important ecological role in marine ecosystems by providing endosymbiotic and metabolic support to coral reefs. Dinoflagellates are eukaryotes with unusual genome compaction strategies that include histone-based chromatinization in addition to DVNPs and HLPs that result in permanently condensed liquid crystalline chromosomes. It is unclear how DVNPs, HLPs, and these divergent histones function in dinoflagellate nuclei. It is known that 5hmU is rare in most eukaryotes but highly abundant in dinoflagellates. The chromatin landscape nor the function of 5hmU are known in the dinoflagellate, B. minutum. It is known that chromosomes adopt three-dimensional distinct domain topology, and this study levies previous Hi-C data to correlate 5hmU and chromatin accessibility.

The manuscript entitled "Genome-wide distribution of 5-hydroxymethyluracil and chromatin accessibility in the Breviolum minutum genome" by Marinov at al. is the first study to report a global map of the distribution of DNA 5hmU and chromatin accessibility in the dinoflagellate species, B. minutum, and adds credence to previous studies that demonstrate 5hmU is abundant in this species. Perhaps surprisingly, the current research did not detect a sequence logos that describes the context of 5hmU that previous studies have found in related species. Instead, the current study reports that 5hmU is enriched in repetitive regions and at topological associated domain (TAD) boundaries but depleted in chromatin accessible regions. Repeat regions tend to be less accessible to ATAC-seq, and there is an anti-correlation between 5hmU and chromatin accessibility. The B. minutum genome is largely accessible, and there does not appear to be any correlation between putative nucleosome positions and TAD boundaries. But, Maverick DNA elements are enriched near TAD boundaries and for putative nucleosome signals. This study reports that many 5hmU reads mapped within Maverick DNA elements, but this is not an exclusive relationship, as most 5hmU reads and residues are found outside of Maverick DNA elements. The totality of the data/interpretations lead to a model wherein 5hmU may play a role in stabilizing dinoTAD boundaries and promoting chromatinization, but this conclusion is based on descriptive and correlative data rather than direct evidence.

The manuscript is received and reviewed with mixed feelings: while many figures could be improved, the methodology and statistical analyses appear to be done quite well. While the analyses are complete, the impactful nature of the results obtained is not sufficiently described or clear…thus, will the community of readers gain much from the descriptive nature of the potential role of 5hmU? This study appears unwilling or limited in its ability to make conclusive statements concerning 5hmU, TADs, and chromatinization and thus it is difficult to determine its overall impact. In general, the results presented are novel, but the implications/relevance of the findings are not well described. It is likely that a revised and conclusion-focused revision could lead to large improvements.

*Reply: We thank the reviewer for the useful comments and suggestions and have revised the manuscript accordingly.*

Comments

* Figure 2: missing panel E; panels B, F, and G do not appear to be referenced in the text. The text does not describe panels F-H at all. The colors and shapes of the circles mean something (surely) but are not described. Mild/harsh oxidation conditions are not explained in sufficient detail for the meaning to be clear. Care should be taken to review each figure for clarity and complete descriptions.

*Reply: We have revised Figure 2 and edited the text to explain the oxidation conditions.*

* Figure 3D could be improved by aligning the x-axis labels.

*Reply: We have revised Figure 3D.*

* Figure 3D figure legend, "S. cerevisiae" should be italicized.

*Reply: This has been corrected.*

* The statement "These results point to increased protein occupancy and…" should be justified and supported with data. Is there any direct evidence of increase protein occupancy? Could such data be generated?

*Reply: The currently existing data is the ATAC-seq datasets themselves. Orthogonal data of other types does not exist currently, nor can it be generated within reasonable constraints on resource expenditure and within the scope of the current study.*

* grammar - line 3 column 1, "an eukaryote" → "a eukaryote"

*Reply: We thank the reviewer for pointing out this typo and have corrected it.*

* grammar Lines 28 column 1, "In order the map…" → " In order to map…"

*Reply: Corrected.*

* Figure 1D. The logos sequence presentation could be improved for clarity. Consider making the Red line at position 6 a "U" or a "T".

*Reply: Making the position a U is not advisable in our humble opinion because it might confuse readers that we are referring to RNA molecules. We have changed the x-axis to position the modified T at 0, for better clarity.*

* Supplementary Figure 1 and 2, why are there several strong ATAC-seq peaks in the gDNA control? Are these the collapsed repeats? It would be ideal if the collapsed repeat regions were clearly labeled.

*Reply: That is the purpose of the gDNA control shown in those tracks – to identify the collapsed repeats. We have edited the figure legends to explicitly specify this.*

* Figure 1G is perplexing. It is difficult to determine/understand that the gDNA control was replicated at 27 and 34 degrees C, but it is unclear what the ATAC curve is referring to, or why the line is black but the text is red. In Figure 1H, it is similarly unclear what the "ATAC" control is in bar 1.

*Reply: We have edited Figure 1G (now Figure 1H) for better clarity. “ATAC” is not a control, but our reference dataset carried out in exponentially growing cultures, while “27C” and “34C” are part of a heat stress experiment. We have revised the text to avoid potential confusion.*

* Typo -- line 46 column 1, "normal temperature (34 C) condition…" → "normal temperature (27 C) condition…"

*Reply: We thank the reviewer for spotting this error. It has been corrected.*

* Supp. Figure 3, What is peak range? It is currently unclear what the x-axis reports.

*Reply: The x-axis in fact shows “peak rank”, not “peak range” (as indicated in the figure itself). We are not sure what the reviewer’s request for changes is here.*

* "Hematodinium sp. DVNP.5 had a negative effect on growth, as previously reported, but much more strongly so than the other two." Should there be a data shown here to support this statement?

*Reply: We unfortunately no longer have the exact growth parameters from those experiments in a form that can be plotted. We have removed this sentence from the text.*

* Typo - line 58 column 2, "more strongly so than the other two"

*Reply: Corrected.*

* Supp Figure 4: it is unclear what the difference is between panel A and B.

*Reply: They just show different regions in the genome, instead of showing only one snapshot.*

* The manuscript would benefit from a short description of the SFM methodology in the text. Supp. Figure 4 panel C and D mention methylation on the y-axis, and but is not sufficiently explained to allow interpretation.

*Reply: We have expanded the text to better introduce the SMF assay and its specifics.*

* Typo - line 61-62 column 2, "ATAC-see" → "ATAC-seq"

*Reply: This is actually not a typo – we really meant “ATAC-see”, which is a different assay from ATAC-seq. In ATAC-see, a fluorescent version on the Tn5 transposase is used to visualize accessible chromatin in nuclei using microscopy.*

**Reviewer 2**

This manuscript reports a series of experiments aimed at characterizing aspects of the chromatin of the symbiotic dinoflagellate Breviotum minutum. In some ways this is a continuation of the effort to shed light on the long mystery of dinoflagellate chromatin, started by the authors recently with the first genome-wide analysis of chromatin conformation in a dinoflagellate. The present study touches on a key issue, which is the presence of the modified base 5-hmU, which substitutes Thymidine and which function, or roles have remained an open question for decades. The authors use a ChIP-seq approach to determine the distribution of 5-hmU (and an additional, chemical modification method) combined with genome wide ATAC-seq and leveraging the existing annotated genome reference, they analyze the relationships between 5-hmU enrichment with chromatin accessibility, TAD boundaries, unidirectional gene arrays, and transposable elements.

This study is important and welcomed as it provides important clues to start putting together the long standing puzzle of dinoflagellate chromatin architecture. However, as with any "first" that intends to show a path forward, addressing potential flaws and shortcoming is critical to sustain the credibility of the approaches. Below I list comments, questions and suggestions to improve the manuscript before its publication.

*Reply: We thank the reviewer for the insightful comments and suggestions.*

First and foremost, this study will arouse the interest of a lot of people that are not familiar with the latest advances in genomics. For them, it will be hard to follow the arguments without a lot of back and forth between the paper, references and other sources. I strongly suggest that a schematic figure with the main methods employed (mainly MeDIP-Seq, the mapping of chemically converted 5-hmU and ATACseq), be included to facilitate understanding by those who have followed the topic for decades but are not on top of the developments in the field.

*Reply: We have added such a figure, as a new 1A panel to Figure 1.*

General comment on figures: the captions are not useful to understand and interpret the figures. Readers should not be compelled to read the methods and even references to be able to get the main idea out of a figure. Prime example (but not the only one): figure 1D.

*Reply: We have revised and expanded the captions as requested.*

I think that to understand figs. 1A, B, H and F a schematic diagram of the procedure and rationale is required.

*Reply: As mentioned above, we have added an entirely new Figure 1, with schematics for the overall strategy and then in details for all assays used.*

Pg. 3, col. 2 lines 33-44: what is the interpretation? Introduce for the readers the idea under multimapping reads.

*Reply: We have expanded the text to better explain the meaning of and significance of multimapping reads.*

Pg. 4, col 1 line 46: It says 34C both times. This analysis could be more interesting than what's portrayed by the authors. This result shows potentially hundreds of regions differentially accessible between the two temperature regimes. I am curious if there is anything interesting to say there.

*Reply: This was a deeply embarrassing typo that has been corrected now. We thank the reviewer for spotting it and apologize for missing it ourselves.*

Repair the labelling scheme of Fig. 2. Panel letters are messed up.

*Reply: Panel letters have been corrected.*

The experiments of heterologous expression of DVNP in yeast appears as an odd addition to the paper. I don't have a strong objection as there are some interesting tidbits from these experiments that add up and complement the story. However, cell growth data for transformed cultured expressing DVNP is missing.

*Reply: We originally had much larger plans for what the heterologous expression of DVNP in yeast might have revealed, which tied very directly to the interpretation of the actual Symbiodinium data (e.g. we were hoping to be able to estimate absolute occupancy protection levels conferred by DVNPs in a controlled way outside their native environment). However, the results that we obtained in yeast (e.g. DVNPs not disrupting the local chromatin environment all that much) did not allow us to use these datasets for much more than to replicate/add to the previous study.*

*It is in our opinion better for the literature as a whole to have an explicit such replication than to just hide these datasets, because we did not the replication and generate the data, and there is no harm is sharing it. Unfortunately, we do not have the growth data in a form that can be plotted quantitatively. We have thus removed this sentence from the text.*

Perhaps the answer to this is found in the references cited, but I'd like to see a short explanation for why a seemingly generalized oxidation reaction would only result in the conversion of 5-hmU into Cytosines without further effects on other bases (modified or otherwise).

*Reply: We have expanded the text and the figures to introduce in detail, rather than just citing the previously published study, the method used here.*

*In short, 5-hmU is converted to 5-fU, which can then basepair with G, resulting in T-to-C conversions. Unlike 5-hmU, the four conventional bases do not have hydroxymethyl groups (or any hydroxy groups) that can be converted to formyl. In addition, we are specifically only quantifying T-to-C conversion events*

I found the data in supplementary figure 6 too important to be buried there. I suggest make a table with all the numbers and including it in the manuscript. A table (matrix perhaps) that shows not only the numbers for the individual sections in the Venn diagram but also the sums (e.g., all 'low accessibility', all '5-hmU enrichment' and the grand total.

*Reply: We have moved this figure to the main text as a new panel to the existing Figure 3.*

Other questions that I have which I don't think are responded:

How many TADs are there, and how many genes they include (mean and range)?

*Reply: The TADs included essentially all genes, as they tile the genome completely and are defined by and precisely coincide with gene arrays.*

*The domains were previously described in our Hi-C paper (Marinov et al. 2021), and number ∼583. This number has been added to the text.*

How reliable is the analysis of positioned nucleosomes (figs S7, 8)? I'm skeptical this has a real value, but I'm open to be convinced otherwise, and in that case, I'd like to hear some more about these nucleosomes, like where they are, how few or many they are compared to what's found in a similarly sized, "normal" eukaryotic genome.

*Reply: The analysis the reviewer requests is already provided in the manuscript, in which there is a direct comparison with yeast, and where the positioned nucleosomes are located preferentially (e.g. over certain repeat classes) is also shown.*

*Ultimately the question of where nucleosomes are found in the genome can only be fully resolved using ChIP-seq, but for that to be possible, dinoflagellate-specific anti-histone antibodies need to be raised, and they need to be raised against all dinoflagellate histones (because they are so divergent that it is not even known which ones are most likely to be the primary ones, and which ones are context-specific variants, if in fact such a distinction exists at all). This is a substantial effort that is well beyond the scope of the current study.*

**Reviewer 3**

The manuscript "Genome-wide distribution of 5-hydroxymethyluracil and chromatin accessibility in the Breviolum minutum genome" maps the distribution of the unusual 5 hmU modification and the chromatin accessibility in the genome of the dinoflagellate B. minutum. The authors find that the 5-hmU modification is enriched over certain types of repetitive elements and also coincides with the boundaries between convergent gene arrays. The modification is also correlated with chromatin inaccessibility. The authors apply an impressive range of methods to study genome modification and organization of this dinoflagellate and discuss their findings briefly in the framework of other dinoflagellates and the only other group of eukaryotes known to possess higher quantities of the 5-hmU modification, the Kinetoplastida.

The analyses are comprehensive and sound and have resulted in very interesting findings. However, the manuscript would greatly benefit from a more detailed explanation of these findings (for non-specialists in these types of analyses and non-specialists in protists), a more stream-lined introduction and from an, in parts, more comprehensive discussion to make it more accessible and informative for a maximum range of readers.

*Reply: We thank the reviewer for the helpful comments and suggestions.*

Major comments (in order of appearance in the manuscript):

Pages 1-2 (Introduction): the introduction is in parts very detailed but it is often hard to understand why certain information is provided, how it is related to the focus of this work. E.g. what is the importance of the paragraph on 5mC methylation for this manuscript and how is this type of hypermethylation in the investigated Symbiodiniaceae relevant? (page 2, left column, lines 57-62).

Page 3, left column, lines 60-62: How does 5mC modification in mammals relate to the modification analyzed here? See also comment about the Introduction above.

*Reply: Mammalian (and not just mammalian) 5mC CpG methylation is the archetypal DNA modification in eukaryotes and what everyone has as a frame of reference in their minds. Thus it being used here as a comparison.*

Overall, the single paragraphs of the Introduction seem sometimes unrelated to each other and the whole introduction section would greatly benefit if they were better thematically connected - to each other and to the rest of the manuscript.

*Reply: We have revised the Introduction substantially in the revised manuscript, as well as the Discussion.*

There is a long, quite confusing paragraph about Kinetoplastida (that are only later introduced to belong to Euglenozoa) on that same page, suggesting this group will play an important role in the manuscript, however they are only mentioned briefly in the discussion.

*Reply: We have substantially reworked the comparison between dinoflagellates and kinetoplastids in the revised text.*

Page 2, right column, line 40: The authors describe the Kinetoplastida as "parasitic clade" which is not correct, as they contain several free-living genera that are common in various environmental habitats (e.g. bodonids).

*Reply: The reviewer is of course correct (e.g. Bodo and others like it certainly exist)– we have corrected the text to “mostly parasitic clade”.*

Page 3, right column, lines 10-14: the authors state that the method used does not allow for estimation of absolute 5-hmU levels. What is the advantage of the method used here over the method(s) used to analyze the other dinoflagellates for which 5-hmU estimates are available?

*Reply: The estimates of 5-hmU for other dinoflagellates date back to the 1970s and 1980s and involved simply measuring the total amount of 5-hmU versus regular thymine in DNA. The chemical mapping we utilized allows profiling 5- hmU levels along the genome at base pair resolution. The ultimate goal is to profile it both genome-wide and with perfect accuracy, i.e. to know that a given position 5-hmU levels are 0%, 25%, 75% or 100%. To achieve that goal, chemical conversion needs to be 100% efficient. However, currently, it is very far from 100% efficient, thus one can know wher 5-hmU is preferentially localized in the genome, and it is also possible to identify any sequence motifs associated with it, but not to measure absolute modification levels at any given position. We have expanded the text to better clarify these points.*

Page 3, right column, 3rd paragraph: the authors state that the ATAC-seq chromatin accessibility analysis resulted in a peak at short fragment length of ~60 bp. Can the authors expand/speculate on this, why 60 bp? and can they provide the expected fragment lengths for nucleosomal chromatin for comparison (only the length for the subnucleosomal peak is given)?

*Reply: We have added the yeast ATAC-seq fragment distribution for comparison.*

*The 60-bp peak likely does not correspond to specific protection footprints, this is just what one gets when DNA is transposed heavily in the absence of strong physical protection from digestion.*

*In fact, the most prominent peak in eukaryotes is of the same length, and it corresponds to the subnucleosomal fragments originating from within open-chromatin regions.*

Page 7, second paragraph: this two sentences seem contradictory: "We first examined the distribution of annotated repetitive elements (see Methods for details) around dinoTAD boundaries (Figure 3A-C), and found no specific preference at dinoTAD boundaries neither for repeats as a whole, nor for any specific repeat family. However, Maverick DNA elements did exhibit strong enrichment around the edges of dinoTADs." Please specify the intended meaning of these sentences. As it is, they are a bit confusing.

*Reply: We have edited the text to avoid causing confusion.*

Discussion, page 8, right column, first paragraph: how do the authors explain the low (and similar to Symbiodinium microadriaticum) levels of 5-hmU in Prorocentrum (Exuviaella = basionym) cassubica? In that context, it would be also helpful for the readers to know that of all the dinoflagellates listed in the Intro as having been analyzed for 5-hmU, only S. minutum belongs to the Symbiodiniaceae, the rest are free-living species with genomes up to two magnitudes higher. This fact should be mentioned to put the 5-hmU numbers into context and maybe also to discuss whether genome organization in the symbiotic species and free-living species (with these massive genomes) can be expected to be the same or whether the differences may not just be in scale. Related also to the comment above - how comparable are those numbers for 5-hmU levels across all those different studies?

*Reply: We are far from sure the 12% number is actually correct for Exuviaella cassubica/Prorocentrum cassubica, because in our survey of the literature in the same sentence we also cite a figure of 62.8% for Prorocentrum micans. The figure for Exuviaella cassubica is from a very old Rae & Steele 1978 paper, the one for Prorocentrum micans is from Herzog et al. 1982. It is highly unlikely there is such drastic variation in the same genus, but these are the reported numbers in the literature.*

*The model suggested by our data is that 5-hmU is found preferentially over repetitive elements. Thus it would be expected that the species with massive genomes would have more 5-hmU globally (as long as that genome expansion is driven by proliferation of repeats, of course).*

*However, something else that we are currently also far from certain of is that the cited estimates for the huge genome sizes of certain dinoflagellates are correct. This issue will of course be resolved by sequencing eventually, and there are certainly some quite large genomes within dinoflagellates, but there are reasons to think the usually cited values are overestimates in many, if not most cases.*

Minor comments:

Page 1, Intro, first paragraph: mention somewhere that dinoflagellates are unicellular eukaryotes. Readers unfamiliar with this lineage may not know.

*Reply: We have added such mention in the revised manuscript.*

Page 3, right column, line 46: which genome assembly was inspected in the genome browser? Two different assemblies are listed in the methods.

*Reply: In terms of the issue with collapsed repeats, it applies to both the original and the scaffolded assembly, as the scaffolding resulted in chromosomes being put together, but did not resolve the collapsed repeats. We have edited the text to clarify this point.*

Page 3, right column, line 16: typo "B. minitum"

*Reply: We thank the reviewer for spotting this error. It has been corrected.*

Page 4, right column, last sentence: it is a bit confusing why C. glabrata cells were used as control without reading the methods first.

*Reply: We have expanded and clarified on why Candida cells were used.*

Page 8, left column, first paragraph: maybe they authors can define in more detail what "fully open/closed" in this sentence means: "It is possible that the open compartment is fully open and the closed one is fully closed"

*Reply: We have added such a clarification.*

**Second round of review**

**Reviewer 1**

All grammatical and typographical errors were corrected. Modifications to text and figures were made to increase clarity. Per other reviewers' comments, additional figures were included for clarity and text was added for broader context. The authors have adequately addressed my concerns. I do not have additional comments, and I recommend this manuscript for publication.

**Reviewer 2**

Thank you for addressing my comments. I believe the manuscript is good to go.

**Reviewer 3**

The authors have carefully addressed all reviewer comments in the response letter and accordingly made comprehensive changes to the original manuscript. There are a few very small comments left that would make the manuscript even more accessible - if addressed, I recommend the manuscript for acceptance.

Page 9, Discussion, paragraph about Kinetoplastids (sorry, no line numbers available):

"Nevertheless, it is tempting to draw parallels between these initial observations in dinoflagellates and what is known in much more details for in another major lineage of eukaryotes – the important mostly parasitic clade Kinetoplastida" Please add either "Euglenozoa" in bracket or add half a sentence such as "that belong to the lineage Euglenozoa" here, as it is still confusing to read then a few sentences later "These properties are shared with other members of the larger Euglenozoa lineage" without mentioning it first. Many people who will read this manuscript for its excellent chromatin analysis of one very unusual lineage may not be familiar with either of the terms.

Page 9, Discussion, 2nd paragraph:

"Alternatively, chromatin structure in basal and core dinoflagellates (Symiodiniaceae belonging to the latter) may differ substantially."

Typo in Symbiodiniaceae. Also, this is on me as I missed it in the first round - the non-core dinoflagellates (Syndiniales and other Marine Alveolates, etc) do not have the typical dinokaryon, supporting the argument that the authors make about fundamentally different chromatin structures. If this can be included, that would strengthen the discussion even more.

Just a nitpick and it is resolved in the manuscript itself. The response of the authors to my first comment about the relevance of 5mC modification as reference to the modification analyzed here "Mammalian (and not just mammalian) 5mC CpG methylation is the archetypal DNA modification in eukaryotes and what everyone has as a frame of reference in their minds." suggests that EVERYONE thinks in the frame of model organisms - the ones that were mostly investigated for 5mC and the associated enzymes. Recently, a few protists were included into these investigations but the full breadth of eukaryotic diversity has not been assessed as far as I am aware (please correct me if I'm wrong, I'd be happy to know about such studies). I'm not claiming that it is not the ancestral methylation, but to say that everyone knows/understands the current distribution of 5mC versus 5-hmU as to understand 5mC as the logical reference in the original manuscript version seems a little optimistic. Nevertheless, the authors have changed the manuscript text in question so that EVERYONE now can quickly understand why 5mC is mentioned.

**Authors Response**

**Point-by-point responses to the reviewers’ comments:**

**Reviewer 3**

The authors have carefully addressed all reviewer comments in the response letter and accordingly made comprehensive changes to the original manuscript. There are a few very small comments left that would make the manuscript even more accessible - if addressed, I recommend the manuscript for acceptance.

*Reply: We thank all three reviewers for their highly useful and constructive feedback and comments.*

Page 9, Discussion, paragraph about Kinetoplastids (sorry, no line numbers available):

"Nevertheless, it is tempting to draw parallels between these initial observations in dinoflagellates and what is known in much more details for in another major lineage of eukaryotes – the important mostly parasitic clade Kinetoplastida" Please add either "Euglenozoa" in bracket or add half a sentence such as "that belong to the lineage Euglenozoa" here, as it is still confusing to read then a few sentences later "These properties are shared with other members of the larger Euglenozoa lineage" without mentioning it first. Many people who will read this manuscript for its excellent chromatin analysis of one very unusual lineage may not be familiar with either of the terms.

*Reply: We have added the requested clarification.*

Page 9, Discussion, 2nd paragraph:

"Alternatively, chromatin structure in basal and core dinoflagellates (Symiodiniaceae belonging to the latter) may differ substantially."

Typo in Symbiodiniaceae.

*Reply: We have corrected the typo, and thank the reviewer for spotting it.*

Also, this is on me as I missed it in the first round - the non-core dinoflagellates (Syndiniales and other Marine Alveolates, etc) do not have the typical dinokaryon, supporting the argument that the authors make about fundamentally different chromatin structures. If this can be included, that would strengthen the discussion even more.

*Reply: We have added this clarification to the Discussion section.*

Just a nitpick and it is resolved in the manuscript itself. The response of the authors to my first comment about the relevance of 5mC modification as reference to the modification analyzed here "Mammalian (and not just mammalian) 5mC CpG methylation is the archetypal DNA modification in eukaryotes and what everyone has as a frame of reference in their minds." suggests that EVERYONE thinks in the frame of model organisms - the ones that were mostly investigated for 5mC and the associated enzymes. Recently, a few protists were included into these investigations but the full breadth of eukaryotic diversity has not been assessed as far as I am aware (please correct me if I'm wrong, I'd be happy to know about such studies). I'm not claiming that it is not the ancestral methylation, but to say that everyone knows/understands the current distribution of 5mC versus 5-hmU as to understand 5mC as the logical reference in the original manuscript version seems a little optimistic. Nevertheless, the authors have changed the manuscript text in question so that EVERYONE now can quickly understand why 5mC is mentioned.

*Reply: We fully agree with the reviewer on the need to move away from mammalian-centric thinking and to comprehensively expand the coverage of the tree of life (it is one of the major goals of our own research). There are indeed more protists that have been/are being profiled, and also, the now standard use of long read single-molecule platforms (PacBio/ONT) for genome sequencing, which also read out methylation directly, will make it possible to provide such coverage in the future.*
